# Lignin Extraction from Various Biomass Sources: A Comprehensive Review of Characteristics, Applications, and Future Prospects

**DOI:** 10.1002/tcr.202500045

**Published:** 2025-06-19

**Authors:** Alaa A. Dandash, Basim Abu‐Jdayil, Joy H. Tannous

**Affiliations:** ^1^ Department of Chemical and Petroleum Engineering United Arab Emirates University F1 Building, P.O. Box: 15551 Al‐Ain United Arab Emirates; ^2^ National Water and Energy Center United Arab Emirates University P.O. Box: 15551 Al Ain United Arab Emirates

**Keywords:** biomass wastes, extractions, green solvents, lignins, lignocellulose

## Abstract

Various biomass resources generate significant byproducts, including lignin, an aromatic polymer known for its abundance, affordability, and functional diversity. Converting lignin into valuable products is essential for a sustainable circular economy. This review discusses the extraction and utilization of lignin from various biomass sources, addressing the different methodologies for its extraction, including physical, chemical, physiochemical, and biological pretreatment. Additionally, its potential applications in biofuels, chemicals, and polymers are explored. The importance of lignin's origin, chemical modifications, and physical characteristics in determining its suitability for different applications is emphasized. This review explores various pretreatment techniques, emphasizing deep eutectic solvent pretreatment for its efficiency in lignin dissolution and depolymerization into valuable aromatic compounds. The review discusses the applications of these advanced pretreatment technologies that can significantly contribute to the sustainable development of lignin applications in biofuels and biochemicals, reducing reliance on fossil fuels and promoting the utilization of renewable resources. Overall, this review is an overview of the lignin extraction processes used for diverse biomass sources, their efficiency, and their implications for downstream applications. It also highlights the versatility and adaptability of lignin extraction techniques across different biomass resources.

## Graphical abstract

Lignin, a major byproduct of various agricultural waste, plays a key role in advancing a sustainable circular economy. There are several challenges faced during lignin extraction due to its complex structure and strong bond in the lignocellulosic biomass matrix. This review emphasizes the impact of lignin's origin and extraction technique on its characterization and potential applications.

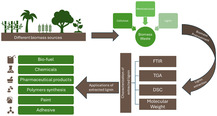

## 1 Introduction

**Figure 1:**
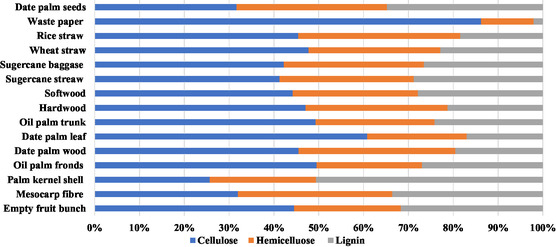
Concentrations of cellulose, lignin, and hemicellulose in different LCB resources.

Over the past decade, there has been increased awareness of the detrimental environmental consequences associated with fossil fuels, including the pollution of air and water, along with their role in greenhouse gas generation.^[^ ^1^ ^]^ Additionally, the depletion of conventional energy resources due to excessive exploitation and the finite nature of fossil fuel reserves has further emphasized the need for alternative energy sources. Consequently, there has been a growing interest in exploring diverse bio‐based chemical constituents for their incorporation into various chemical compounds, polymers, and novel biofuels for commercial purposes as a more sustainable alternative to fossil fuels. Lignocellulosic biomass (LCB) has captivated the attention of researchers as it is the most abundant organic matter on Earth, with a worldwide yearly production of ≈1.3 × 10^10^ metric tons annually.^[^ ^2^ ^]^ LCB comprises primarily cellulose, hemicellulose, and lignin, with variable proportions of these polymers depending on the different plant species involved, as cited in various research works^[^ ^3^, ^4^, ^5^, ^6^, ^7^, ^8^, ^9^ ^]^ and illustrated in **Figure** **1**.

**Figure 2:**
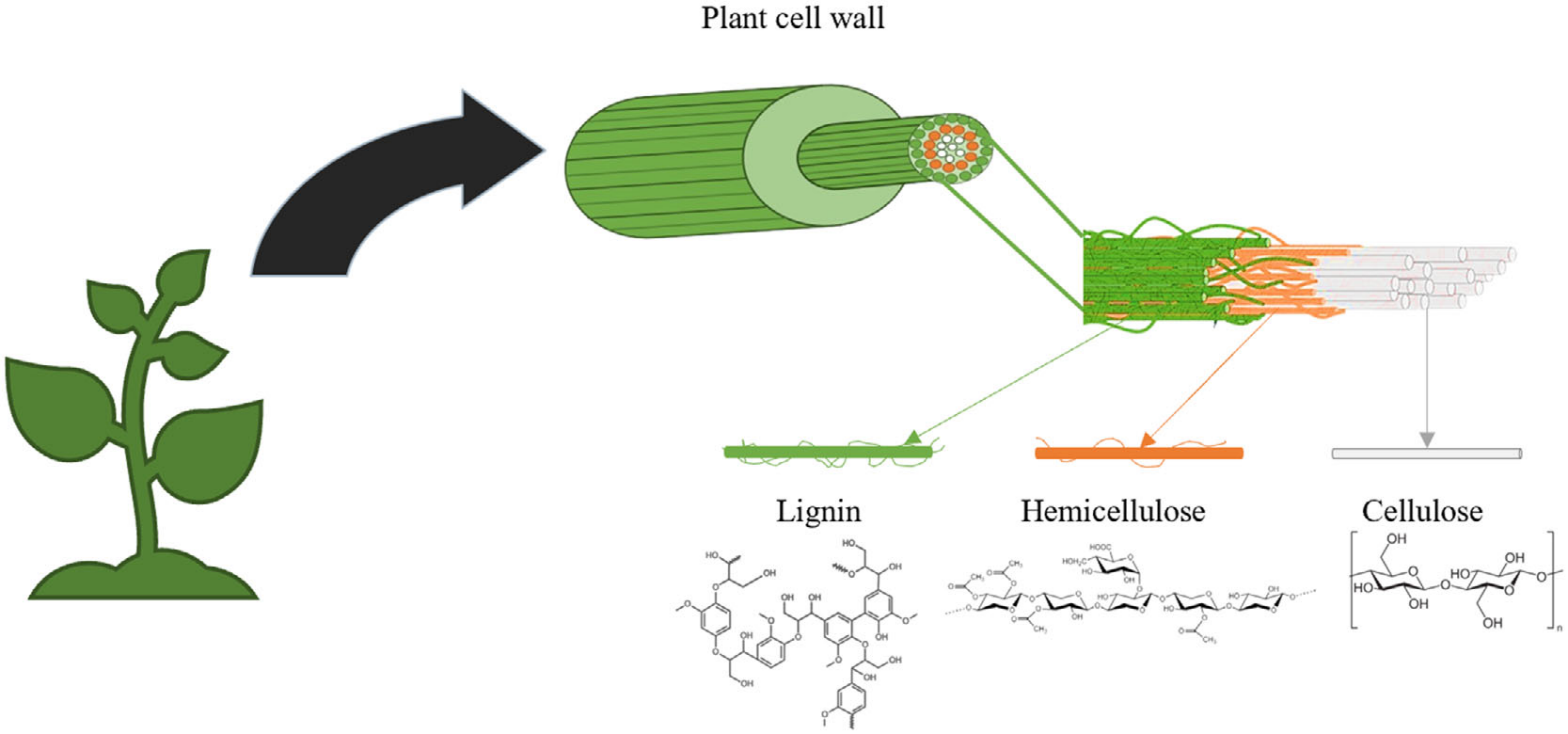
LCB matrix components include cellulose, lignin, and hemicellulose.

The intricate LCB matrix illustrated in **Figure** **2** consists of cellulose enveloped by a monolayer of hemicellulose and embedded within a matrix of hemicellulose and lignin, rendering the separation of lignin from the cell wall and cellulose challenging during isolation processes.^[^ ^10^ ^]^ Figure 2 shows the components of LCB and its structure, in which cellulose is a linear polymer comprising *β*‐D‐glucose units linked by *β*‐1,4‐glycosidic bonds. The chains form straight, rigid structures that align parallel to each other. Cellulose provides tensile strength and rigidity to plant cell walls. Hemicellulose is a heterogeneous group of polysaccharides consisting of various sugar monomers such as xylose, mannose, and galactose. Hemicellulose functions as an interstitial material that connects cellulose microfibrils, enhancing their cohesion and flexibility. Additionally, it enhances the formation of chemical bonds between cellulose fibers and the integration of lignin. Lignin is a complex, irregular polymer composed of phenolic compounds that provide compressive strength, rigidity, and resistance to microbial degradation.^[^ ^11^ ^]^

**Figure 3:**
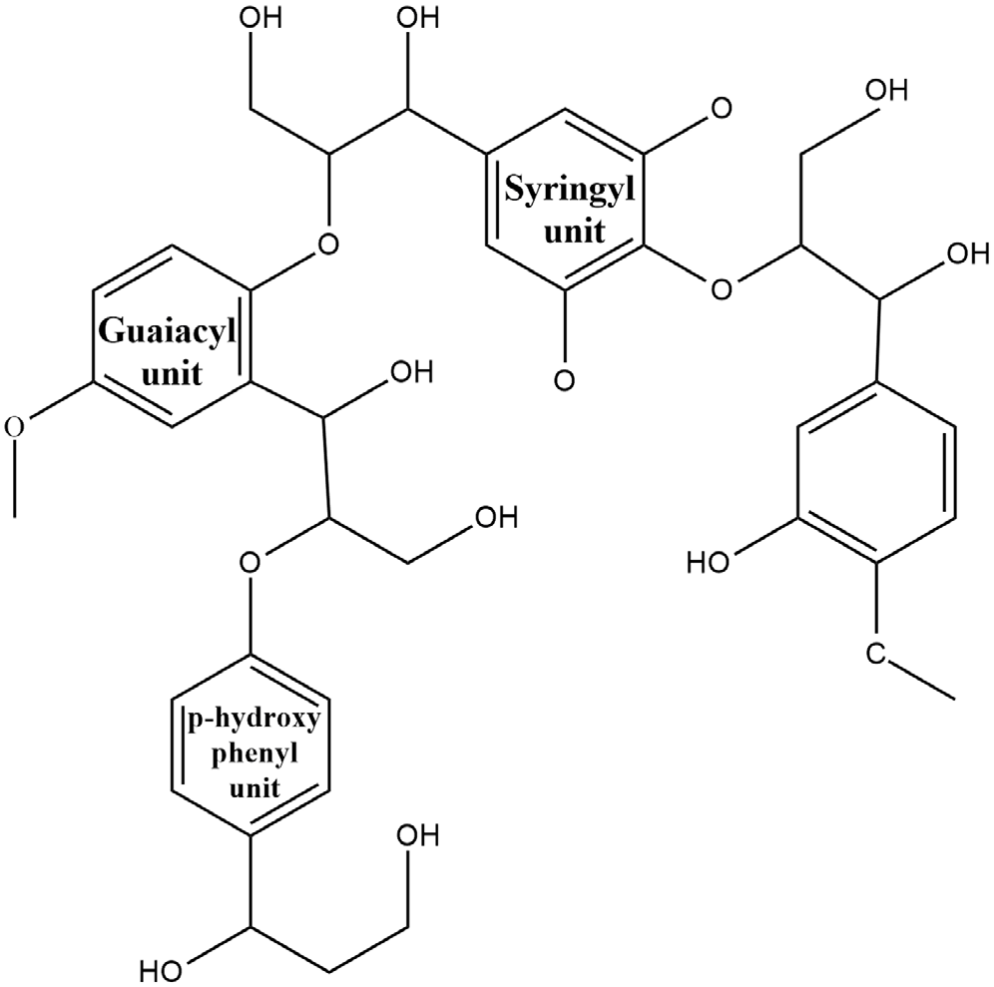
Chemical structure of lignin.

Historically, the main goal of lignocellulosic pretreatment was to boost enzymatic cellulose hydrolysis by releasing polysaccharides from the lignocellulosic matrix, with potential hemicellulose and lignin extraction and utilization attracting little attention. However, in recent times, the importance of utilizing the components of lignocellulose for various product streams has been emphasized due to the growing emphasis on sustainable development and reducing reliance on fossil fuels.^[^ ^12^ ^]^ The separation of cellulose was discussed and reviewed by many researchers,^[^ ^7^, ^13^, ^14^, ^15^, ^16^ ^]^ leaving lignin as an abundant unutilized byproduct. Lignin is a naturally occurring aromatic polymer and contains three types of phenylpropanoids comprising *p*‐hydroxyphenyl, guaiacyl, and syringyl units, which are interconnected through ether bonds and carbon–carbon bonds,^[^ ^17^ ^]^ as illustrated in **Figure** **3**. Lignin, a significant byproduct of the pulp and paper industry, is characterized by its abundance, affordability, sustainability, and the presence of diverse functional groups such as aromatic and hydroxyl groups. Its hydrophilic nature, biodegradability, antioxidant properties, and reinforcing potential make it an attractive option for producing bioaromatic chemicals such as vanillin and phenols, bio‐based materials such as resins and polymers, and carbon fibers. Moreover, lignin can be utilized as reinforcement fillers in thermoplastic polymers or as dispersants.^[^ ^18^ ^]^ Lignin separation and purification can be challenging depending on the potential applications and the source of biomass.

The first step in lignin utilization is its extraction from biomass. Researchers have investigated various lignocellulosic pretreatment methods for lignin extraction from biomass, including physical, chemical, physio‐chemical, and biological processes, which are extensively utilized to convert diverse biomass materials into biofuels and value‐added compounds.^[^ ^19^, ^20^ ^]^ The second section of this review paper will generally discuss the different pretreatment methods and their mechanisms, advantages, and disadvantages.

**Figure 4:**
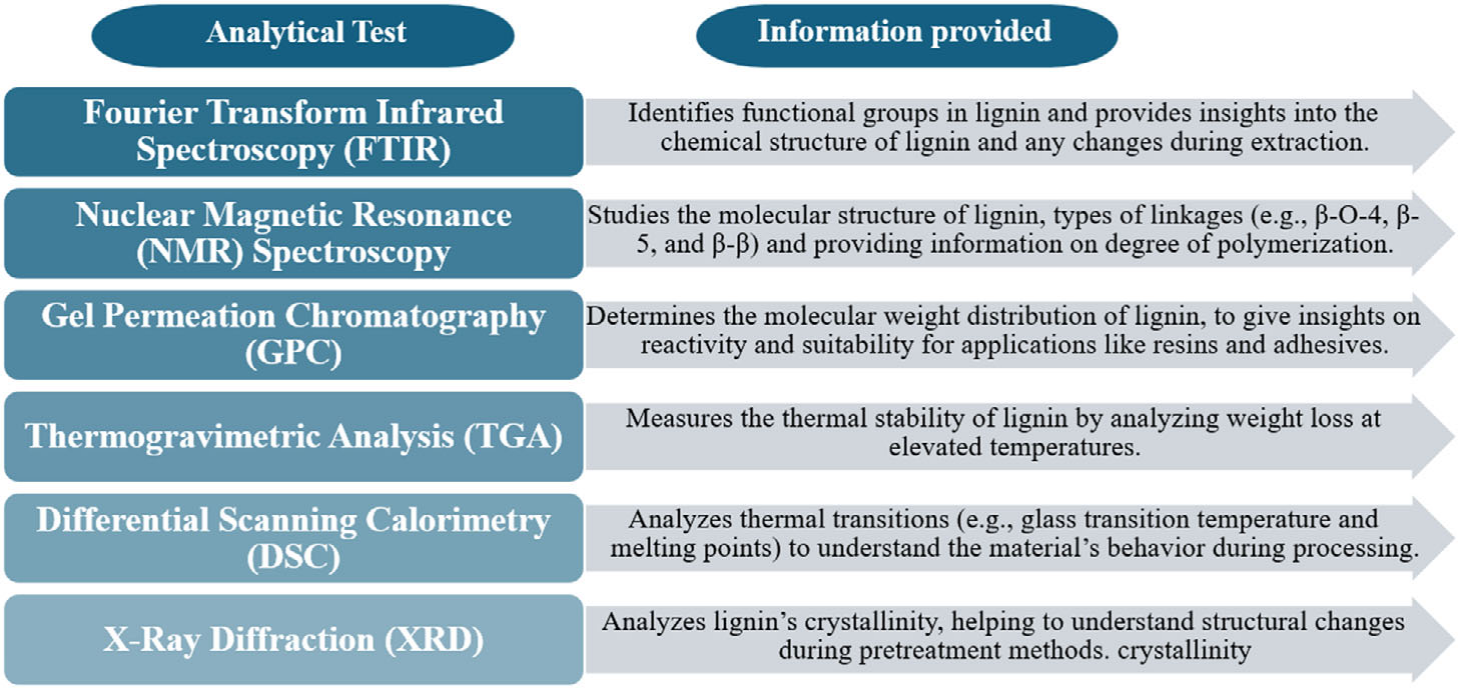
Common analytical lignin characterization and their respective purposes.

The methodologies used for isolation vary depending on the raw material utilized as the biomass source, as well as the characteristics of the lignin extracted; for example, isolation procedures from wood may diverge from those employed for green plant parts such as leaves and stems.^[^ ^21^, ^22^ ^]^ In light of these differences, the current study aims to provide an in‐depth review of lignin extracted from various biomass and its utilization in various applications. The various biomass sources, the pretreatment methods used for lignin extraction from each biomass, the characteristics of the lignin extracted, and its potential applications will be covered. **Figure** **4** summarizes the common analytical tools for lignin characterization and their respective purposes. The third section aims to emphasize the range of biomass materials, the specific extraction methods suited to each, and the potential applications of the extracted lignin.

Nonetheless, lignin's heterogeneous polymeric structure, inherent recalcitrance, distinctive odor, dark color, high bond dissociation enthalpies in its functional groups, and other factors pose challenges in its valorization. Lignin can be used throughout various applications that include biofuels, chemicals, and polymers, among others. Recent research has studied lignin as a valuable resource in medicinal applications, particularly drug delivery. However, the effectiveness of these applications relies on many factors, such as the origin of the lignin, its chemical changes, and its physical and chemical characteristics.^[^ ^23^ ^]^ Based on these insights, the fourth section of this review focuses on lignin applications not covered in the third section, offering a broader perspective on lignin's potential applications across diverse fields.

Although previous review articles have discussed lignocellulosic pretreatment methodologies,^[^ ^22^, ^24^, ^25^, ^26^, ^27^ ^]^ they have several limitations, including the lack of a thorough comparison of novel green solvents, such as deep eutectic solvents (DESs) and new organic solvents such as cyrene and 1,4‐butanediol, which are becoming more environmentally friendly and effective in lignin extraction. Previous reviews also neglected the significant impact of biomass origin on lignin structural properties and potential applications. This study addresses these issues and evaluates how biomass source variability affects the extracted lignin's properties, which can affect its suitability for high‐value applications such as biofuels, bioplastics, and chemicals. This review's novelty and unique contributions lie in presenting the most recent advancements in lignin extraction methods over the past four years. Furthermore, this review emphasizes the significance of lignin's origin, investigating how different biomass sources influence the extracted lignin properties and potential applications, which is an important aspect that has received limited attention in prior reviews.

This work will introduce a comprehensive and in‐depth review of lignin extraction from diverse biomass sources, focusing on the advancements in pretreatment methods and highlighting the innovations that have enhanced the yield, purity, and physical characteristics of lignin extracted. Furthermore, this study aims to underscore the potential of lignin as a valuable resource for biofuels, chemicals, and materials as well as its contribution to the broader goal of sustainable development, reducing dependency on fossil fuels, and fostering a circular bioeconomy. Additionally, the review aims to highlight the gaps in the literature regarding the extraction of lignin from various biomass resources, providing insights into challenges and opportunities for future research.

## 2 LCB Pretreatment Techniques

The conversion of LCB into nonenergy products is achievable through the separation of its primary components, such as lignin and cellulose, using various techniques. These methods include physical, chemical, and combined physical–chemical pretreatments, as well as biological pretreatment. Such processes facilitate the dissolution and extraction of lignin and cellulose, enabling their subsequent application in diverse fields. These techniques will be covered in this section, and a comparison between different LCB pretreatment methods. Physical pretreatment comprises the size reduction of raw material by mechanical techniques to assist in further chemical treatment. The selection of a suitable treatment approach depends on the intended application of the extracted lignin and the particular biomass employed. It is important to recognize that various extraction methods yield different types of lignin, including Klason lignin, kraft lignin, organosolv lignin, alkali lignin, hydrolytic lignin, and milled wood lignin. The extraction procedure significantly impacts both the structural framework and the molecular weight distribution of the extracted lignin obtained from different biomass sources.^[^ ^28^ ^]^ Many reviews discussed the different LCB pretreatment methods and their advantages and disadvantages.^[^ ^25^, ^29^ ^]^

### 2.1 Physical Pretreatment

**Figure 5:**
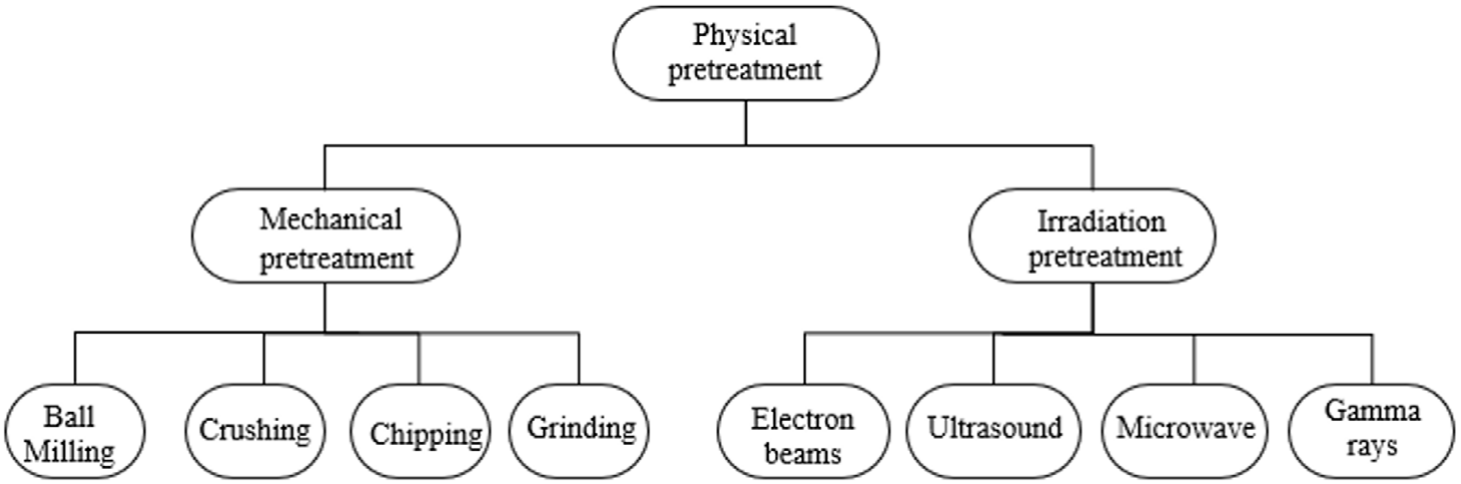
Physical pretreatment methods of LCB.

Many physical pretreatment methods are used to achieve diverse objectives, including reducing molecular size, enhancing storage capacity, reducing moisture content, and homogenizing feedstock, to address the complex structure of biomass. Physical pretreatments change biomass‐derived material qualities such as specific surface area, crystallization index, and polymerization degree. Common physical pretreatment methods include mechanical processing and irradiation,^[^ ^30^ ^]^ as shown in **Figure** **5**.

### 2.2 Chemical Pretreatment

**Figure 6:**
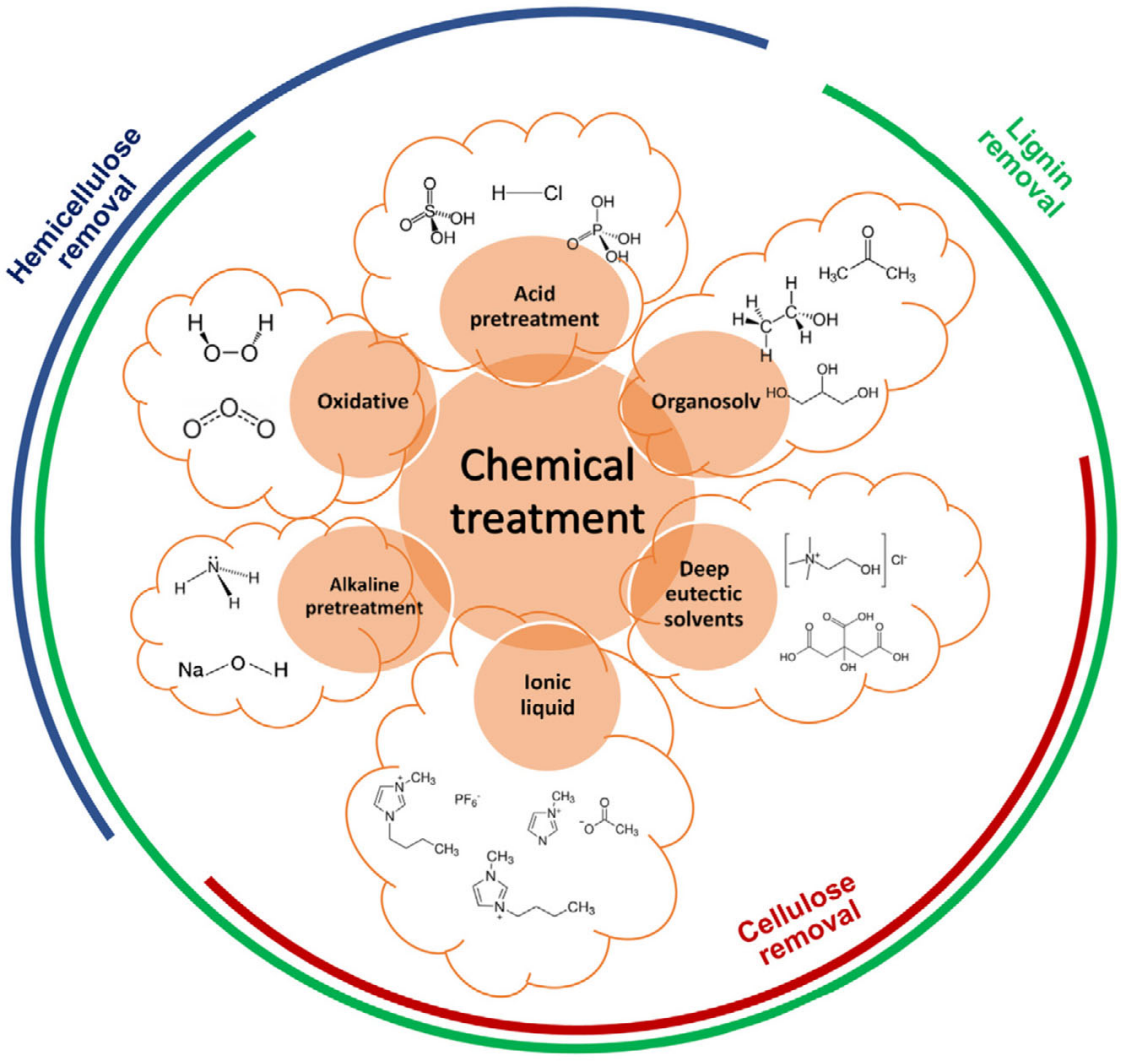
Visual presentation of different methods of chemical pretreatment. Reproduced with permission.^[^ ^32^ ^]^ Copyright 2019, Elsevier.

**Figure 7:**
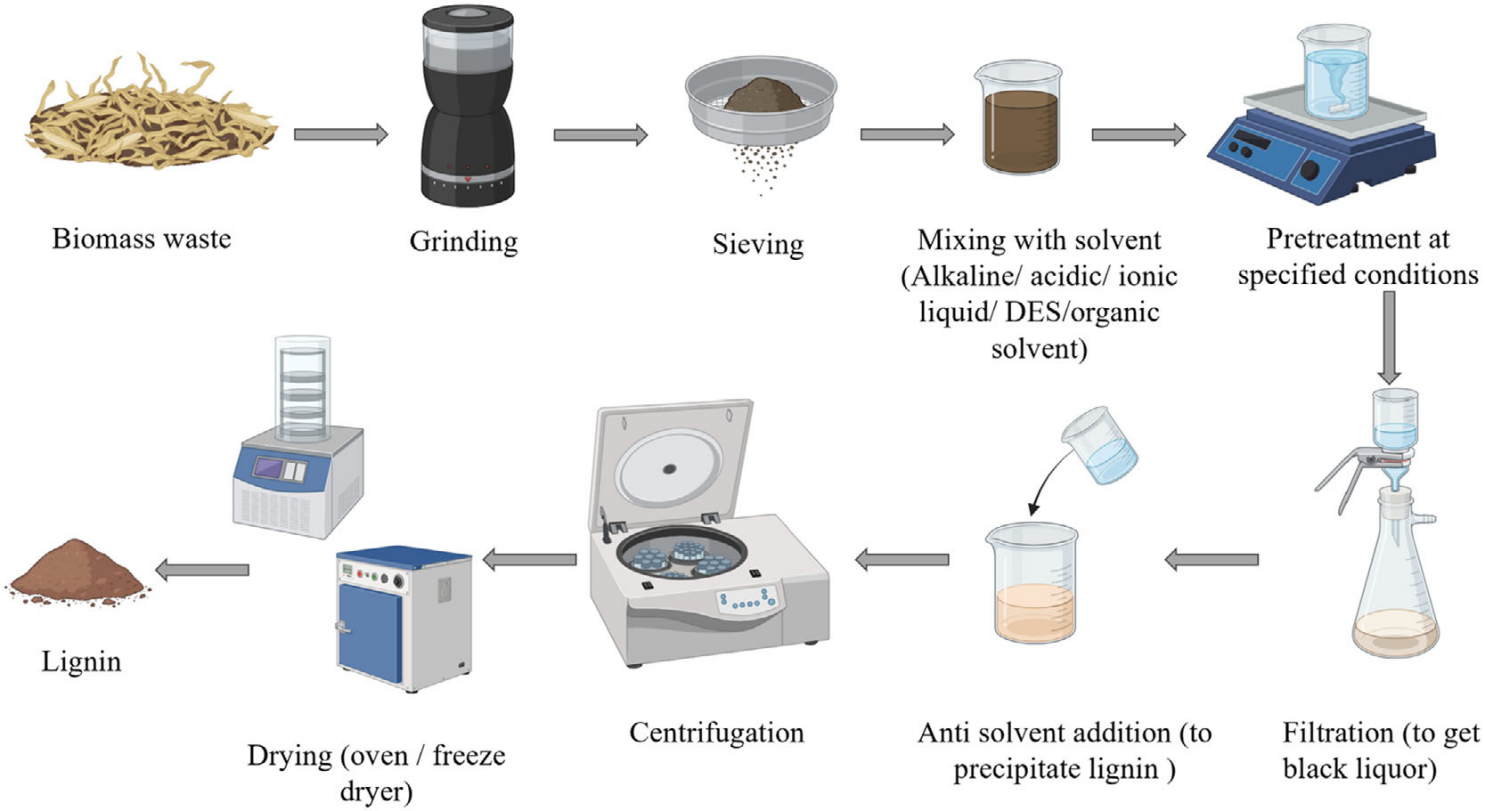
Flow diagram for lignin's chemical extraction method.

Chemical pretreatment is frequently employed to extract the biopolymeric components of LCB: lignin, cellulose, and hemicellulose. Pretreatment involves introducing either organic or inorganic solvents in order to disrupt the biomass framework by interacting with the intrapolymer and interpolymer interactions of the initial organic biomass components. During chemical pretreatment, the inherent durability of the LCB structure is altered, leading to reduced cellulose crystallinity, depolymerization, cellulose degradation, and lignin breakdown.^[^ ^31^ ^]^ There are several chemical pretreatment techniques utilized in various industries, including acidic, alkaline, organosolv, ionic liquid (IL), and DESs pretreatment.^[^ ^19^ ^]^ The choice of a specific treatment method and the steps involved largely depend on the application in which the extracted cellulose, hemicellulose, or lignin will be used, as illustrated in **Figure** **6**.^[^ ^32^ ^]^ Chemical pretreatment is considered the most commonly used pretreatment method for lignin extraction. Moreover, occasionally these methods can be assisted by physical pretreatment approaches to minimize chemical usage and enhance the overall efficacy of the pretreatment.^[^ ^33^ ^]^ Lignin's chemical extraction method consists of several significant steps, as shown in the flow diagram in **Figure** **7**. Under specified treatment circumstances, the biomass is first ground and then mixed with a solvent—an IL, DES, alkaline, acidic, or organic solvent. Filtration then separates the black liquor, including lignin, from the cellulose fraction following mixing. The black liquor is then mixed with an antisolvent to precipitate the lignin, which is then centrifuged and dried.

#### 2.2.1 Acidic Pretreatment

Within the array of pretreatment techniques, acid treatment emerges as a notably efficient method for dissolving hemicelluloses, which enhances cellulose accessibility and leaves a higher concentration of cellulose and lignin in the pretreated biomass. Acid pretreatment primarily functions by cleaving aryl ether linkages, notably *β*‐*O*‐4 linkages, within lignin, resulting in partial lignin depolymerization and removal, as well as hydrolysis of hemicellulose.^[^ ^34^ ^]^ This process employs both inorganic acids (e.g., sulfuric acid, nitric acid, phosphoric acid, and hydrochloric acid) and organic acids (e.g., formic acid, acetic acid, oxalic acid, and propionic acid).^[^ ^35^ ^]^

#### 2.2.2 Alkaline Pretreatment

Alkaline pretreatment relies on dissolving lignin in alkaline solutions. As lignin is capable of creating soluble complexes known as lignin chelates with metal cations under alkaline conditions, it can be dissolved in an alkaline solution.^[^ ^36^ ^]^ Bases such as NaOH, KOH, Ca (OH)_2_, and NH_3_ are commonly used in alkaline pretreatment of lignocellulose biomass. Each agent has unique benefits and considerations based on biomass feedstock, desired outcomes, and process conditions.^[^ ^37^ ^]^ Alkaline pretreatment of biomass increases biomass surface area and decreases cellulose crystallinity and polymerization. This technique makes the biomass structure more accessible to bacteria and enzymes, enabling biological or enzymatic reactions.^[^ ^38^ ^]^ Alkali pulping is the most common chemical pulping technology in the pulping and papermaking industries, and alkali lignin and its derivatives are used in petroleum, chemical, environmental protection, coating, and ceramics applications.^[^ ^39^ ^]^

#### 2.2.3 Organosolv Pretreatment

The organosolv biomass pretreatment process dissolves hemicellulose and extracts lignin using pure or composite organic solvent solutions combined with, for example, water, alkali, or acid. This procedure usually happens at 120–250°C, and uses ethanol, acetone, methanol, ethylene glycol, or tetrahydrofurfuryl alcohol. Bases such as ammonia, sodium hydroxide, and lime, or mineral acids like sulfuric, hydrochloric, and phosphoric acid, can aid organosolvation, and certain salts can catalyze these reactions.^[^ ^37^ ^]^ Organic solvents are commonly categorized into two types: polar protic and aprotic organic solvents. Polar protic solvents, including methanol, ethanol, and ethylene glycol, can break lignin–hemicellulose, ether, and glycosidic bonds, allowing lignin oligomers and oligosaccharides to solubilize, and preventing repolymerization. In contrast, polar aprotic solvents such as dimethyl sulfoxide (DMSO) can solubilize LCB without breaking chemical bonds due to their restricted ionization.^[^ ^20^ ^]^ Organosolv pretreatment produces high‐quality lignin, which is appropriate for biomaterials due to its purity, sulfur‐free nature, and low functional group changes.^[^ ^40^ ^]^ However, solvent recovery and recycling can reduce solvent costs and their detrimental effects on microorganism growth, enzymatic hydrolysis, and fermentation. Due to their flammability, strong organic solvents must be handled carefully to avoid serious damage and fire explosions.^[^ ^33^ ^]^

#### 2.2.4 Ionic Liquid Pretreatment

In recent times, ILs have emerged as a viable option for the pretreatment of LCB owing to their beneficial attributes, including high customizability, reusability, low volatility, and superior separation capabilities, among others. ILs have also garnered recognition as green solvents. These liquids, characterized by organic salts comprising cations and anions, are able to effectively degrade LCB into its subcomponents.^[^ ^41^, ^42^ ^]^ Notably, ILs possess distinct physicochemical properties, including thermal stability, nonflammability, ease of recovery, and melting points below 100 °C,^[^ ^43^ ^]^ rendering them highly desirable for replacing flammable and volatile solvents in various applications.

The dissolution and extraction of lignin in ILs involve the disruption and reorganization of hydrogen bonds within LCB. Anions play a pivotal role in solubilizing lignin by forming hydrogen bonds with it. The interaction force between the IL and lignin surpasses that between lignin molecules, facilitating lignin dissolution in the IL. However, the interaction force between IL and water exceeds that between IL and lignin, which allows water to act as an antisolvent, weakening or breaking the hydrogen bonds between IL and lignin, thereby causing lignin precipitation

ILs cause disruption and reorganization of hydrogen bonds within LCB, aiding the dissolution and extraction of lignin. Anions create hydrogen bonds with lignin to solubilize it. Lignin dissolves in the IL because its interaction force with IL is greater than that of lignin's molecules.^[^ ^44^ ^]^ There are still certain obstacles that remain relevant in the utilization of ILs as a pretreatment approach, including the high costs involved with ILs, as well as the complexity of the recovery and reuse of ILs after pretreatment. Additionally, the lack of process technologies that facilitate the efficient utilization of ILs must be further investigated.^[^ ^45^ ^]^

#### 2.2.5 DESs Pretreatment

A deep eutectic solvent (DES) consists of a hydrogen bond acceptor (HBA) and a hydrogen bond donor (HBD), forming a mixture with a melting point significantly lower than that of its individual components. Typically, DESs are created by combining a quaternary ammonium salt with a metal salt that forms a complex with its halide ion.^[^ ^46^ ^]^ This unique property of DESs allows them to effectively separate lignocellulose by breaking the ether linkages between lignin and carbohydrates.^[^ ^47^ ^]^ The strong hydrogen bond interactions between the HBA and HBD within the DES facilitate the release of low‐molecular‐weight lignin.^[^ ^48^ ^]^ The procedure of extracting lignin from plant biomass using a DES, first, the plant biomass powder is mixed with the DES and heated to the target temperature; after the reaction, the resultant liquid is filtered. Antisolvent is added to the soluble fraction to precipitate and purify lignin, then the precipitated and purified lignin is dried.^[^ ^49^ ^]^ DES can replace ILs for biomass pretreatment, leading to reduced costs, as DES production is simpler than that of ILs and uses inexpensive, commonly available materials. DESs also have other benefits, such as low volatility, wide liquid range, low toxicity, biodegradability, and enzyme compatibility.^[^ ^50^ ^]^

### 2.3 Biological Pretreatment

Biological pretreatment of LCB for lignin extraction entails employing microbes or enzymes to alter the structure of lignocellulose, making it suitable for lignin separation. This technique commonly involves the activity of fungi, bacteria, or specialized enzymes to break down or alter the intricate composition of lignocellulose, improving the ease of accessing and extracting lignin from the biomass matrix.^[^ ^51^ ^]^ Biological pretreatment provides a sustainable and environmentally friendly method for extracting lignin, minimizing the requirement for harsh chemicals and energy‐intensive procedures, while optimizing the use of LCB.^[^ ^52^ ^]^ **Table** **1** presents the different LCB pretreatment methods and their mechanism, advantages, and disadvantages.

**Table 1:** Comparison between different LCB pretreatment methods. Reproduced with permission.^[^ ^20^, ^29^ ^]^

	Type	Mechanism of pretreatment	Advantages	Disadvantages
Physical pretreatment	Mechanical pretreatment	Increases the surface area by reducing the biomass's size	Decrease in particle size and cellulose's crystallinity	High Power consumption, additional processing necessary, poor yield
	Pyrolysis pretreatment	The thermal decomposition process breaks down the complex organic compounds present in LCB	Depolymerize the lignin and hemicellulose components of LCB, enzyme hydrolysis of cellulose is easier	High‐energy use, complex mixtures of products, including undesired compounds such as tar and char
	Irradiation pretreatment	Destruction of chemical bonds	Simple, reliable, no other reagents need to be added	Necessity of additional pretreatment steps, sometimes high costs for large‐scale treatment
Chemical pretreatment	Acidic pretreatment	Elimination of lignin, hydrolysis of cellulose and hemicellulose fibers	Quick separation, high hydrolysis rates	Corrosion of equipment, high cost
	Alkaline Pretreatment	Reduces cellulose polymerization, decomposes hemicellulose, and removes lignin	Increased surface area and porosity, reduction in cellulose crystallinity	High‐cost, acid neutralization is required after the reaction
	Organosolv pretreatment	Extracts lignin and hemicellulose from LCB, improves enzyme hydrolysis of cellulose	High‐purity lignin, Solvent recovery and reusability, relatively mild conditions	High cost of solvents, the possibility of environmental pollution
	Ionic liquid pretreatment	Disruption and reorganization of hydrogen bonds within LCB	Recovery with antisolvent, environmental protection, ionic liquids can be tailored to target specific biomass	Ionic liquids can be relatively expensive, recovery and reuse of ILs are complex
	Deep eutectic solvents	Disrupts the structure of biomass components, solubilizes lignin and hemicellulose from the biomass	Nontoxic, biodegradable, starting components to produce DESs are available and inexpensive	DESs may have high viscosity leading to longer processing times, limited scalability
Biological pretreatment	Bacteria	Lignin and hemicellulose removal	Mild reaction conditions, low energy use, zero pollution, effective adaptation to high temperatures and pH	Low efficiency and lengthy processing, many bacteria cannot be cultivated in a lab
	Fungi	Decomposes lignin	High level of degradability	Strains grow slowly, prolonging treatment, the strain may consume cellulose and hemicellulose
	Enzymes	Hydrolyzes cellulose, hemicellulose, and lignin	Ability to target substrates for easier access	High cost

## 3 Lignocellulosic Biomass Sources for Lignin Extraction

Widely readily available sources of LCB include palm tree wastes, corn stover, rice husks, rice straw, (SCB), and coconut husk.^[^ ^53^ ^]^ The second‐most abundant natural polymer in LCB is lignin, accounting for 10%–25% of the biomass,^[^ ^54^ ^]^ which is distinguished by its stability and insolubility in water, where it functions as the “adhesive” that bonds cellulose and hemicellulose. The following section will review research done to investigate lignin extraction using various pretreatment techniques from palm trees, sugarcane, bamboo, rice waste, and other lignocellulosic biomasses. **Table** **2** presents the percentage composition of the three major components of the biomass resources discussed in the upcoming section, along with the elemental composition of each component. Additionally, lignin, across different biomass sources, has different structural properties. The H:G:S (*p*‐hydroxyphenyl:guaiacyl:syringyl) ratio of lignin significantly influences its structural properties and potential applications. Bagasse‐derived lignin, with a high syringyl content (H:G:S = 2:38:60), exhibits a high proportion of *β*‐*O*‐4 ether linkages, enhancing its solubility and reactivity, making it suitable for biopolymers, bio‐based polyurethanes, antioxidants, and fine chemicals. In contrast, straw lignin is guaiacyl‐rich (H:G:S = 4:68:28), leading to a more condensed and crosslinked structure, which improves thermal stability and makes it ideal for phenolic resins, adhesives, and carbon fibers.^[^ ^55^ ^]^ Bamboo lignin (H:G:S = 1:15:34) presents a balanced composition, offering moderate solubility and structural rigidity, making it suitable for thermoplastic composites, sustainable coatings, and biomedical applications.^[^ ^56^ ^]^ These variations in lignin composition underscore the importance of selecting an appropriate biomass source based on the targeted application, particularly in bio‐based material development.

**Table 2:** Percentage composition and elemental analysis of major components in different biomass resources.

Biomass resource	Cellulose %	Hemicellulose %	Lignin %	Ref.
Elemental composition	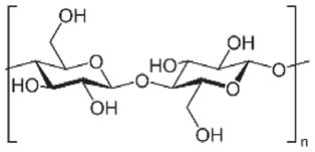	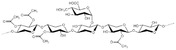	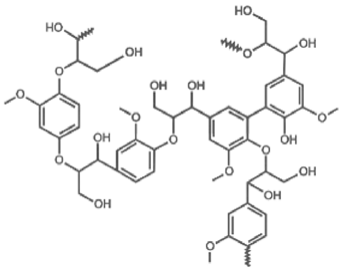	
Date palm leaves	41.90	29.50	21.40	[141]
Date palm wood	39.8	30.6	17.1	[7]
Sugarcane bagasse	42.40	25.20	19.7	[142]
Bamboo	45.78	26.56	23.37	[143]
Rice straw	34.7	29.3	19	[144]

### 3.1 Extraction of Lignin from Palm Tree Waste

Palm trees, which are widely cultivated across tropical and subtropical regions, represent a diverse group of species with significant potential as sources of lignin. Among them, oil palm (*Elaeis guineensis*), date palm (*Phoenix dactylifera*), coconut palm (*Cocos nucifera*), and sugar palm (*Arenga pinnata*) are of particular interest due to their abundant biomass production, as there are more than 2,500 palm tree species, which can produce over 1,000 products.^[^ ^57^ ^]^ These palms, especially date palm and oil palm trees, generate large quantities of lignocellulosic residues, including empty fruit bunches, fronds, trunks, and husks, which are typically underutilized. The lignin content in these residues varies depending on the palm species and part of the plant, but generally, they are rich in lignin, cellulose, and hemicellulose, making them promising candidates for lignin extraction.

**Figure 8:**
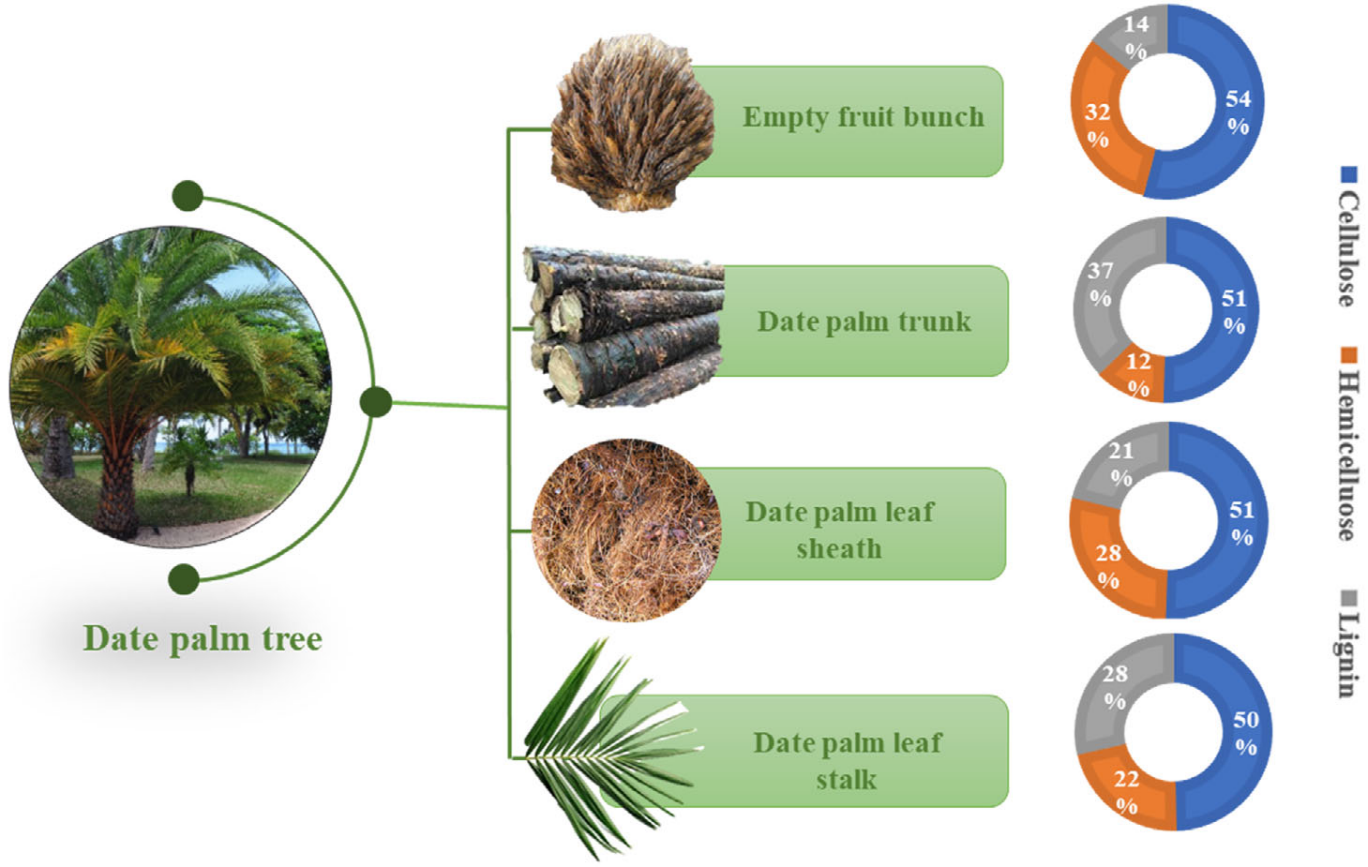
Different date palm wastes and their compositions.

Despite the lack of water, date palm trees grow to produce fresh fruit, rendering them well‐suited for desert environments like the UAE, with an actual population of date palms of ≈ 40 million. Further, date palm oil is extracted from the mesocarp of the fruit, resulting in large agricultural wastes such as dry leaves, fronds, pits, seeds, and fruit remnants.^[^ ^9^ ^]^ Typically, these date palm leftovers are either burned on farms or disposed of in landfills, leading to environmental damage. The solid waste that results from the palm oil industry represents one of the greatest sources of lignocellulosic solid waste globally, topping 12 million metric tons annually. Consequently, there exists potential to convert these wastes into higher‐value goods such as biofuels, chemicals, and biomaterials.^[^ ^58^ ^]^ **Figure** **8** illustrates different date palm wastes and their compositions.^[^ ^59^ ^]^

To fully exploit the potential of this vital resource, advanced practical approaches for extracting lignin from various date palm biomass sources are being studied, including chemical, physical, and combination treatments. Several studies published in the past few years have focused on lignin extraction from palm tree waste, which will be discussed further in the following section. **Table** **3** summarizes some of the research done to extract lignin from palm trees, showing the different applications where the extracted lignin was utilized.

**Table 3:** Overview of studies for the lignin extraction from palm tree waste.

Biomass	Method	Procedure	Yield	Properties	Application	Ref.
Date palm kernel	Acidic treatment. (Klason method)	Two major steps. One is to remove the extractives from the DPW with ethanol–benzene to make them extractive‐free (waxes, oils, resins, gums), while the other is to treat the residue obtained by sulfuric acid.	26.68%	hydroxyl groups at peaks at wavenumber around 3350 cm^−1^. Lignin incorporation improved the Membrane's porosity and hydrophilicity; as membrane porosity reached 37%, the contact angle decreased by 28 %	Low‐density polyethylene (LDPE) polymeric membrane modification.	[60]
Leaflet			22.54%			
Pedicel			25.47%			
Palm frond			31.63%			
Fibrilium			29.22%			
Oil palm empty fruit bunch fiber (OPEFBF)	Alkaline treatment	Aqueous sodium hydroxide and ammonia solutions treatment was conducted via different to investigate optimum conditions, then vacuum filtration to separate treated OPEFBF from the black liquor.	33.0 ± 2.2%	Optimum condition (at 1:7 OPEFBF‐to‐ammonia, 50 °C and 96 h), about 64.0 ± 0.4% of klason lignin removed. NH_3_ lignin showed intensified bands in the 1590‐1220 cm^−1^ region, indicating aromatic structures. However, the band intensity for NaOH‐lignin suggests that it is carbohydrate‐contaminated.	–a)	[63]
The date palm tree fronds	Alkaline treatment (kraft and soda pulping)	For Kraft pulping, a mixture with 20% active alkali and 30% sulfidity was applied, using a water‐to‐fiber ratio of 10. In soda lignin pulping, a 30% w/v concentration of NaOH was utilized at 150 °C for 4 h.	17 ± 0.2% kraft lignin 21 ± 0.1% Soda Lignin	Good thermal stability, KL and SL (DTG_max_) at 375 °C and 381 °C, indicating SL had higher thermal stability compared to KL. KL had higher phenolic—OH amount (1.4/Ar) than SL(.9/Ar)	Kraft lignin phenol glyoxal (KLPG) and soda lignin phenol glyoxal (SLPG) resins	[64]
Black liquor of oil palm empty fruit bunches (EFB)	Acidification and Physical mechanical treatment (ball milling)	H_2_SO_4_ precipitation for black liquor. Further purification of lignin as the lignin powder was stirred in (C_2_H_5_)_2_O to remove fats and fatty acids. Lignin nanoparticles LNPs were fabricated using high‐energy ball milling.	–a)	Lignin nanoparticle sizes range from 46 nm to 80 nm and 45 nm to 67 nm. As 5 % lignin was incorporated in the prepared bioplastic films an increase in contact angle of 43.29% indicating more hydrophobicity.	Biodegradable plastic films	[66]
Oil palm empty fruit bunch	Organosolv pretreatment	Ethanol ‐ water Mixture, along with NaOH as a catalyst. Subsequently, the addition 20% H_2_SO_4_ to precipitate lignin	13.7%	64.5% lignin content with hydroxy phenolic content of 6.8% indicating potential to be utilized as a natural antioxidant for biodiesel.	Biodiesel antioxidant	[67]
Oil palm biomass (trunk‐ frond – empty fruit bunch)	Ionic liquid	Ionic liquid [bmim][Cl] Followed by lignin precipitation either by AlK(SO_4_)_2_‐12H_2_O or acidified H_2_SO_4_ or acidified HCl	OPT (19.05 ± 1.17 − 22.78 ± 1.47) OPF (15.52 ± 0.71 −19.08 ± 1.83) OPEFB (9.50 ± 1.09 − 11.6 ± 1.07)	low Mw lignin not exceeding 1500 g/mol. Depending on the source, OPT, OPF, and OPEFB had degradation temperatures of 215, 207.5 and 272°C.	–a)	[70]
Oil palm empty fruit bunch fiber (OPEFBF)	Deep eutectic solvent	Choline chloride: lactic acid [CCLA], glucose: lactic acid [GLUCLA], choline chloride: D (+)‐glucose [CCGLUC], choline chloride: glycerol [CCGLY], choline chloride: urea [CCUREA], and potassium carbonate: glycerol [K2CO3] GLY]	88% delignification and 50% lignin pellet extraction	Acidic DES was found to have the largest biopolymer dissolution capacity	–a)	[72]

a) Information was not reported in the cited literature.

Five types of date palm waste (DPW), namely date palm kernel, leaflet, pedicel, palm frond, and fibrilium derived from palm trees located in the oasis of Gabes, Tunisia, were investigated for lignin extraction. Initial characterization of the raw DPW used found that fibrilium and palm fronds had higher lignin content. Zrelli^[^ ^60^ ^]^ investigated lignin extraction using the Klason method, an acidic pretreatment recognized for its simplicity, cost‐effectiveness, and quick extraction process.^[^ ^61^ ^]^ After crushing DPW, extractives were extracted using an ethanol–benzene mixture to remove waxes, oils, and gums, followed by 72% sulfuric acid treatment. The liquid was diluted to 3% H_2_SO_4_, filtered, and dried. Lignin yields ranged from 31.63% to 22.54%, with palm fronds yielding the most, followed by fibrilium, date palm stone, pedicel, and leaflet, yielding the least at 22.54%. Fourier‐transform infrared spectroscopy (FTIR) Spectroscopy indicated a broad band around 3350 cm^−1^ observed for all samples, indicating a high concentration of hydroxyl groups. Total phenolic hydroxyl groups ranged from 0.018 mmol/g for palm frond lignin to 0.572 mmol g^−1^ for date palm kernel lignin; the variations in phenolic content and functional group concentrations observed by UV–vis spectroscopy underscore the dependency of the lignin extracted on the DPW source, influencing its reactivity and utility in polymer modifications. The recovered lignin was added to a polymeric solution to make low‐density polyethylene (LDPE) membranes using the inversion phase process. Due to its numerous hydroxyl groups, isolated lignin altered the characteristics of the membranes. The study highlighted the enormous potential of lignin as an additive to LDPE membranes for treating oily wastewater.^[^ ^60^ ^]^ The Klason method is considered advantageous due to its fast reaction and high extraction yield, but the problem of equipment corrosion, leading to higher costs, encouraged researchers to investigate other pretreatment techniques to extract lignin.

To further investigate the efficiency of lignin extraction from oil palm empty fruit bunch fibers (OPEFBF) supplied by Seri Ulu Langat Palm Oil Mill Sdn Bhd, Malaysia, an alternative approach was employed, involving alkaline pretreatment with aqueous sodium hydroxide (NaOH) and aqueous ammonia solutions. Alkaline pretreatment was commonly reported as the most effective treatment in delignification.^[^ ^62^ ^]^ Aqueous alkaline medium saponifies intermolecular ester linkages that cross‐link carbohydrate and lignin in biomass, enabling lignin extraction from the pretreatment biomass to maximize the waste value. FTIR research showed that NaOH‐lignin was severely polluted with carbohydrate fragments, whereas high‐temperature, short‐time NH_3_‐lignin was the purest. The most effective pretreatment method for lignin extraction involved the lowest temperature, longest‐replenishment approach. Under optimal conditions (1:7 OPEFBF‐to‐ammonia ratio, 50 °C, and 96 h), ≈64.0 ± 0.4% of Klason lignin was removed from the OPEFBF, and 330.3 ± 21.9 mg g^−1^ of lignin was successfully recovered from the black liquor with minimal structural degradation.^[^ ^63^ ^]^ In an investigation of the potential applications of lignin extracted from date palm fronds (DPF) collected from date palm trees in Abu Dhabi, UAE, in December 2018, they were subjected to alkali pretreatment for lignin extraction. The extracted lignin was then evaluated as a potential alternative to phenol in the production of LPG resins. DPF powders were pulped using both kraft and soda processes to extract lignin. The kraft pulping solution had 70% active alkali and 30% sulfidity, whereas soda lignin pulping employed NaOH. The extracted lignin was characterized to determine its suitability as a phenol alternative in LPG resins. Kraft and soda lignin from date palm fronds had differential thermogravimetric (DTG_max_) curves with maximum values of 375 °C and 381 °C, respectively. In adhesive preparation, extracted kraft and soda lignin replaced phenol at various ratios. Compared to 50% soda SLPG and PG resins, the 50% kraft lignin phenol glyoxal (KLPG) glue had the highest solid content (40%), viscosity (0.165 Pa‐s), and gel time (140 s). Compared to DPF resin (58.57 MPa), the 50% (w/w) KLPG resin (65.3 MPa) was stronger. KLPG resin has more phenolics than soda lignin, which may explain this result. Its strength and nontoxicity make the 50% KLPG resin a viable green wood glue alternative to DPF adhesives.^[^ ^64^ ^]^ Another promising application of lignin is its use as a reinforcement in bioplastic films.^[^ ^65^ ^]^ In this context, lignin nanoparticles (LNPs) were extracted and purified from black liquor obtained from oil palm empty fruit bunches (EFBs) through a soda pulping process, provided by the Division of Bioresource Technology at Universiti Sains Malaysia. To precipitate lignin from EFB black liquor, H_2_SO_4_ was gradually added dropwise until the pH reached 2.0. The lignin was then filtered, rinsed with water, and further purified by (C_2_H_5_)_2_O to remove fats and fatty acids, and then washed and dried. Both impure and purified LNPs were then high‐energy ball milled. The purified LNPs were solution‐cast into *Kappaphycus alvarezii* bioplastic sheets at different loadings, aiming to improve their characteristics. Bioplastics saw improvements in shape, surface roughness, structure, hydrophobicity, water barrier, antibacterial characteristics, and biodegradability. The films were proven to be effective in inhibiting bacterial growth against gram‐positive and gram‐negative bacteria. Pure LNP films outperformed impurified LNPs, with bioplastic films with 5% pure LNPs having the best characteristics. After 40 days of soil burial, all bioplastic films were totally biodegradable, suggesting they might replace fossil fuel‐based plastics in packaging materials for various uses.^[^ ^66^ ^]^ Another notable application of lignin is its use as an antioxidant in biodiesel production. Alcell commercial lignin and oil palm empty fruit bunch (EFB) powder, supplied by Balai Besar Teknologi Pati (B2TP‐BPPT) in Lampung Tengah, Indonesia, were tested for their antioxidant activity in biodiesel. The organosolv pretreatment method extracted lignin from EFB by mixing equal parts water and 95% ethanol with NaOH as a catalyst in an autoclave reactor. Organosolv pretreatment yielded 13.7% lignin with a lignin purity of 64.5%. Both organosolv and commercial lignin were added to biodiesel at concentrations of 500, 1000, and 1500 ppm. This study used BHT as a positive control. Biodiesel oxidation stability was assessed using the Rancimat test, which showed that 1500 ppm organosolv lignin induced biodiesel in 4.2 min, similar to 1000 ppm commercial lignin and 500 ppm BHT, promoting the use of commercial and organosolv lignin from OPEFB as biodiesel antioxidants.^[^ ^67^ ^]^

Recently, there has been an emphasis on employing environmentally friendly solvents such as ILs and DESs as substitutes for volatile organic solvents. ILs are regarded as green alternatives because they do not generate hazardous chemicals upon application. Additionally, IL can be feasibly recovered and reused.^[^ ^68^ ^]^ ILs can be customized by modifying the branching and length of the alkyl chains linked to the cation. Tailoring ILs in this manner enables their design to play a significant role in the efficient breakdown of LCB.^[^ ^69^ ^]^ In another study, lignin was extracted and characterized from various oil palm biomass (OPB) sources, including oil palm empty fruit bunch (OPEFB), trunk (OPT), and frond (OPF) waste collected from a farm in Johor, Malaysia. First, biomass was dissolved in 1‐butyl‐3‐methylimidazolium chloride ([bmim][Cl]), followed by CO_2_ gas purging and addition of aluminum potassium sulfate dodecahydrate (AlK(SO_4_)_2_‐12 H_2_O) to remove lignin from lignin‐rich IL solution. Another precipitation method that used H_2_SO_4_ and HCl solutions to precipitate lignin obtained the highest lignin yield (22.78%) from an oil palm trunk using AlK(SO_4_)_2_ – 12 H_2_O) for lignin precipitation. The yield was in order OPT > OPF > OPEFB, corresponding to their lignin content. FTIR spectra confirmed the extraction of homogeneous lignin from OPB. Various OPB types had various molecular weights, which affected their thermal degradation. Mohtar et al. recommended using environmentally friendly IL and nontoxic aluminum potassium sulfate dodecahydrate for lignin precipitation to maximize ecological integrity and process efficiency.^[^ ^70^ ^]^

DESs utilization in biomass processing represents a noteworthy transition toward more environmentally friendly procedures, prompting DES‐lignin isolation research.^[^ ^71^ ^]^ This unique property of DESs allows them to effectively separate lignocellulose by breaking the ether linkages between lignin and carbohydrates.^[^ ^47^ ^]^ The strong hydrogen bond interactions between the HBA and HBD within the DES facilitate the release of low‐molecular‐weight lignin.^[^ ^48^ ^]^ Lignin was isolated from oil palm EFB from a plantation in Bera, Pahang, Malaysia, using six DESs ranging from acidic to basic. Synthesized DESs were used in fractionation and delignification for oil palm EFB. DESs were made from materials with diverse functional groups. The six DESs investigated included Choline chloride: lactic acid, glucose, D(+)‐glucose, glycerol, urea, and potassium carbonate. EFB was uniformly mixed with DES at 120 °C. Organic acid‐based DES demonstrates considerable potential in biomass processing. Remarkably, choline chloride: lactic acid [CCLA] achieved 100% hemicellulose extraction, 88% delignification, and 50% lignin recovery from EFB, followed by pretreatment with glucose: lactic acid [GLUCLA], which had a similar mass distribution.^[^ ^72^ ^]^

In summary, among the different approaches used for palm tree waste, alkaline pretreatment yielded the highest lignin recovery, with up to 94% from oil palm residues, but resulted in some contamination with carbohydrates, reducing its purity and limiting its applicability in specific fields. In contrast, DESs demonstrated a lower lignin recovery (50%–88%) but offered high purity and better environmental compatibility. The lignin extracted using DESs, especially with choline chloride: lactic acid (CCLA), achieved 88% delignification and 50% lignin recovery, making it suitable for high‐value applications such as bioplastics and biofuels. Additionally, studies should focus on further optimization of pretreatment parameters to increase the yield of lignin extracted using DESs.

### 3.2 Extraction of Lignin from Sugarcane Bagasse

The global production of sugarcane amounts to ≈ 1.6 billion tons annually.^[^ ^73^ ^]^ In the current global scenario of sugarcane production, Brazil is the leading producer, with an annual production of ≈ 739,300 metric tons, followed by India, China, Thailand, Pakistan, Mexico, Colombia, Indonesia, the Philippines, and the United States.^[^ ^74^ ^]^ The sugarcane industry generates several byproducts, including sugarcane straw, the harvest residue, and sugarcane bagasse (SCB), the fibrous material left after juice extraction. Together, these byproducts account for ≈279 million metric tons of SCB annually.^[^ ^75^ ^]^ The substantial quantity of sugarcane biomass, particularly bagasse, deserves attention due to its potential environmental impact if left unaddressed; moreover, these byproducts, specifically bagasse, which is considered a valuable feedstock for various applications, owing to its chemical composition. Bagasse is known to be rich in cellulose (32%–44%), hemicellulose (27%–32%), and lignin (19%–24%), and contains a certain percentage of ash (4.5%–9%).^[^ ^76^ ^]^ Given the principles of a sustainable circular economy, converting these sugarcane industry byproducts into value‐added products holds significant importance. In the following section, a summary of existing research on the extraction of lignin from sugarcane lignocellulosic materials will be provided. This process typically involves a pretreatment step to disrupt the structure of lignocellulose and separate lignin. **Table** **4** summarizes the literature discussed in this section, the different properties and applications where the extracted lignin was utilized.

**Table 4:** Overview of studies for the lignin extraction from sugarcane waste.

Biomass	Method	Procedure	Yield	Properties	Application	Ref
Sugarcane bagasse	Alkaline treatment	Pre‐treated with hot water (70°C) and alkaline aqueous solutions (15% of sodium hydroxide (NaOH), 98°C). Sulfuric acid was utilized to acidification to precipitate lignin.	13%	Higher molecular weight (2781 g/mol) and good thermal stability (180 °C) High reactivity due to the high content of hydroxyl group indicated by Band 3400–3405 cm^−1^.	Lignin–phenol‐formaldehyde wood adhesive.	[77]
Sugarcane bagasse	Alkaline treatment	Pretreatment using sodium hydroxide dialysis for the removal of alkali	–a)	Total phenolic content (69.41 ± 0.32 mg gallic acid equivalent/g extract), antioxidant activity (262.30 ± 2.98 mg) Trolox equivalent/g extract), and sun protection factor (8.65±0.21). Offering UVA and UVB‐absorbing properties and tyrosinase‐inhibitory properties.	Multifunctional ingredient that can offer antiageing, sun‐protection, and skin‐whitening properties for sun care formulations.	[79]
Sugarcane bagasse	Organosolv	Solvent mixture comprising MIBK/methanol/water (35%: 25%: 40% v/v) with various concentrations of H_2_SO_4_ catalyst	87% of lignin recovery	Lower Mw (1374 g mol_1) and polydispersity index (1.75) compared to commercial organosolv lignin. DTG_max_ of 437 °C higher than commercial lignin indicating thermal stability.	–a)	[80]
Sugarcane bagasse	Acidic pretreatment	Acetic acid	55%	The presence of phenolic hydroxyl groups identified by peaks at 1215–1227 cm^−1^, increases the reactivity of lignin Moreover, the maximum degradation temperature of acetosolv lignin was higher than that of Kraft lignin.	Phenol‐formaldehyde resin	[78]
Sugarcane bagasse	Organosolv	Aqueous ethanol (70% v/v) at various temperatures	Recovery yield of 65.3%w/w	All lignin samples extracted have higher absorption for UVB (290–320 nm) than UVA (320–400 nm). Lignin treated at 230 °C had Antioxidation property of 12.50 ± 1.96 (μg/mL) and SPF value 17.32 ± 0.02 (5%w/v)	Sunscreen	[82]
Sugarcane bagasse	Ionic liquid	Ethanolamonium acetate Ethanolamonium formate Ethanolamonium lactate Ethanolamonium malonate Ethylenediamonium acetate Ethylenediamonium formate Ethylenediamonium lactate Ethylenediamonium malonate	–a)	The amination process, occurs naturally during extraction evidenced by NMR signal enhancement observed in the A_ɣ_ (59.0/3.4 ppm) after using [Ac] anions IL.	–a)	[81]
Acidic sugarcane bagasse	Deep eutectic solvents (DES)	ChCl‐Gly ChCl‐Urea	lignin removal 81.1%	An increase in temperature and reaction time favors the removal of lignin	–a)	[83]

a) Information was not reported in the cited literature

Delignification of SCB from a Doukkala, Morocco facility by alkaline pretreatment was investigated, using sodium hydroxide‐extracted biolignin for plywood adhesive applications. First, the bagasse was pretreated with hot water to dissolve hemicelluloses. After pretreatment, the bagasse was cooled, washed, and centrifuged. The pretreated bagasse was then immersed in 15% NaOH to extract lignin. After filtration, sulfuric acid was used to acidify the black liquor to a pH of 2, precipitating the lignin, which was then centrifuged and air‐dried. Dry weight was used to calculate lignin and cellulose yields of 13% and 42%, respectively. The recovered lignin powder from sugar cane bagasse had high reactivity due to its high hydroxyl group content, additionally, its molecular weight of 2781 g mol^−1^ and heat resilience to 180 °C made it a promising PF resin alternative. The extracted lignin was used to make lignin‐PF wood adhesives without further processing. Lignin and PF were copolymerized at room temperature to make lignin‐PF adhesives. Five‐ply plywood panels were made with bagasse lignin‐PF resin bonding plywood. The plywood was hot‐pressed in an industrial setting. It was found that lignin can replace 30% of PF resins in plywood bonding without affecting quality. Additionally, lignin‐PF panels emitted less formaldehyde and met global standards for interior‐grade plywood, offering environmental advantages.^[^ ^77^ ^]^

Pinheiro et al. conducted a study aimed at replacing petroleum‐derived phenols in resin production by optimizing the extraction of lignin from SCB and utilizing it for synthesizing phenol‐formaldehyde resin.^[^ ^78^ ^]^ The SCB used was obtained from an alcohol and spirits company in Ceará State, Brazil. Various acetic acid concentrations and an HCl catalyst were used in the acetosolv procedure, where 95% (w/w) acetic acid and 1:20 solid mass per volume of reagent were shown to be optimal. A central composite design determined the optimal temperature and reaction time for SCB lignin extraction. The optimal acetosolv extraction yielded 55% ± 4.5% using 95% (w/w) acetic acid and 0.1% v/v HCl at 187 °C for 40 min. The extracted lignin had stronger thermal stability, a lower molar mass, a higher proportion of *p*‐hydroxyphenyl units, increased phenolic and total hydroxyl content, and lower methoxyl group levels compared to kraft lignin. These acetosolv‐extracted lignins had structural properties suitable for phenol‐formaldehyde resin production. Resins were synthesized using phenol, 37% aqueous formaldehyde, and sodium hydroxide as an alkaline catalyst, with lignin successfully replacing 40% of the phenol.^[^ ^78^ ^]^

Lignin derived from SCB also shows potential for use in antiaging, sun‐protective, and skin‐whitening cosmetic applications. The lignin was extracted through alkaline pretreatment, and its yield was optimized using response surface methodology (RSM), with varying concentrations of sodium hydroxide, temperatures, and reaction times employed for the alkaline hydrolysis of SCB. The extracted lignin was then analyzed for its phenolic content, antioxidant capacity, and sun protection factor (SPF). The optimal extraction conditions for lignin extracts with high phenolic content, antioxidant activity, and SPF were 7% sodium hydroxide, 135 °C, and 47.92 min. The lignin extracts inhibited tyrosinase and absorbed ultraviolet A radiation and ultraviolet B radiation light. These findings implied that lignin extract could be used to make antiaging, sun‐protective, and skin‐whitening cosmetics.^[^ ^79^ ^]^ Another central composite design‐based RSM was utilized to optimize an acid promoter for organosolv extraction of lignin from SCB from Mae Chan, Chiang Rai, Thailand. Methyl isobutyl ketone (MIBK), methanol, water, and H_2_SO_4_ catalyst were combined with screened SCB in a stainless steel reactor. Following the pretreatment, the mixture was filtered, and the cellulose‐rich solid fraction was dried at 105 °C. The chemical composition of the separated solid, including cellulose yield, hemicellulose removal, and lignin recovery, was analyzed using the National Renewable Energy Laboratory (NREL) method. The optimal experimental conditions were 0.02 M acid, 200 °C, and 90 min in a solvent mixture of MIBK, methanol, and water (35%: 25%: 40% v/v%). 88% of lignin was removed, and 87% was recovered from the organic phase. The study's sulfuric acid‐catalyzed solvothermal pretreatment efficiently extracted high‐value organosolv lignin from SCB with minimal contamination.^[^ ^80^ ^]^

Lignin recovery upon acidic or alkaline pretreatment is considered challenging due to degradation or chemical alteration. Alternatively, organosolv, IL, and DES pretreatment technologies produce high‐quality lignin for many applications while being environmentally friendly. The potential of enhancing sunscreen properties using organosolv lignin extracted from SCB sourced from Petchaboon, Thailand, was investigated. The bagasse was pretreated with ethanol as the organosolv agent and an acid catalyst. The black liquor after filtration was thermally modified in a high‐pressure reactor, and lignin was precipitated with distilled water as an antisolvent. The lignin suspension was described and added to sunscreens. Lignin mixes were mixed with foundation cream or SPF 30 cream, and UV transmittance was evaluated to estimate SPF. Results indicated that thermal treatment increased phenolic hydroxyl content, improving UV absorbance and free radical scavenging in modified lignin. The ultrasonication‐assisted solvent exchange process also created well‐distributed microsphere lignin colloids, boosting the product's sun protection and antioxidant capabilities.^[^ ^81^ ^]^ Eight protic ionic liquids (PILs) were investigated for SCB lignin extraction. Different combinations of two amine cations and four organic anions were used to pretreat eight lignins. Amines were chosen as cations for PIL formulations due to their cost‐effectiveness. Acetic acid, lactic acid, formic acid, malonic acid, ethylenediamine, and ethanolamine were used to manufacture PILs. ILs cause disruption and reorganization of hydrogen bonds within LCB, aiding the dissolution and extraction of lignin. IL's anions create hydrogen bonds with lignin to solubilize it.^[^ ^44^ ^]^ After the PIL treatment, the isolated lignins were characterized to better understand their structure. PIL pretreatment took 2 h with 10% solids loading and a 5:1 IL: H_2_O ratio. The recovered lignins were vacuum‐dried and labeled by PIL. The study revealed that lignins extracted using PILs, especially those with amine‐based components, hold considerable promise for industrial applications due to their high aromatic compound content. Notably, the inherent amination process in PIL extraction eliminates the need for additional processing steps, which are usually necessary for lignins derived from other sources. The structural characteristics of these lignins varied depending on the specific cation and anion components of the PILs, with the extraction temperature significantly influencing the yield. Data analysis of lignins obtained using anion [Lac] PILs suggests promising preservation of the original lignin structure. Conversely, lignins extracted using [2He] [For] and [Etid][Ac] exhibited considerable modifications in their original structure. Particularly, lignin extracted with [Etid][Ac] displayed a marked inclination toward recondensation reactions, evident from its elevated molecular weight (Mw).^[^ ^82^ ^]^ DESs are considered eco‐friendly, affordable, and effective for lignin extraction. By mixing HBA and HBD components, these fluids have strong hydrogen bond interactions, enhancing lignin extraction from LCB. A study focusing on the optimization of lignin extraction from SCB provided by the National Institute for Forestry, Agricultural, and Livestock Research, using DESs. The study examined ChCl–Gly and ChCl–Urea DES variations to determine the effect of temperature and reaction duration on lignin removal. The response surface approach was used to optimize reaction time, liquid–solid ratio, and temperature. The SCB provided has already been acidified (0.86% v/v H_2_SO_4_ for 22.7 min at 121 °C) to remove hemicellulose. Composition study of the carbohydrate‐rich residue (CRR) helped calculate lignin removal percentage. A positive association was found between reaction time, temperature, and lignin removal effectiveness. The surface response approach determined the best conditions for lignin removal from SCB, producing 81.2% and 82% removal rates for ChCl–Gly and ChCl–urea at 160 °C under 23.4:1 and 17:1 LSRs. DES treatment also converted cellulose in SCB from 50% to 80%, demonstrating its lignin extraction ability.^[^ ^83^ ^]^

In conclusion, sugarcane waste, particularly SCB, has considerable potential for value‐added applications. Among the discussed pretreatment methods, alkaline pretreatment using sodium hydroxide achieved a relatively low lignin yield of 13% compared to Acetosolv pretreatment, yielding 55% lignin, yet the alkaline extract was reported to have good thermal stability (180 °C), and high reactivity suitable for wood adhesives. Organosolv extraction using (MIBK/methanol/water) mixture exhibited a superior lignin recovery of 87%, with lower molecular weight (1374 g mol^−1^), improved thermal stability (DTG_max_ of 437 °C), and better structural properties for UV‐protective applications. DES pretreatment was also reported to achieve high lignin removal rates of (81.2%–82%) while preserving lignin quality.

Among these methods, organosolv pretreatment showed the best combination of high lignin recovery, thermal stability, and structural properties, making it the most effective for high‐value applications. Recycling the solvents can further improve the process's economic and environmental sustainability.

### 3.3 Extraction of Lignin from Bamboo

**Figure 9:**
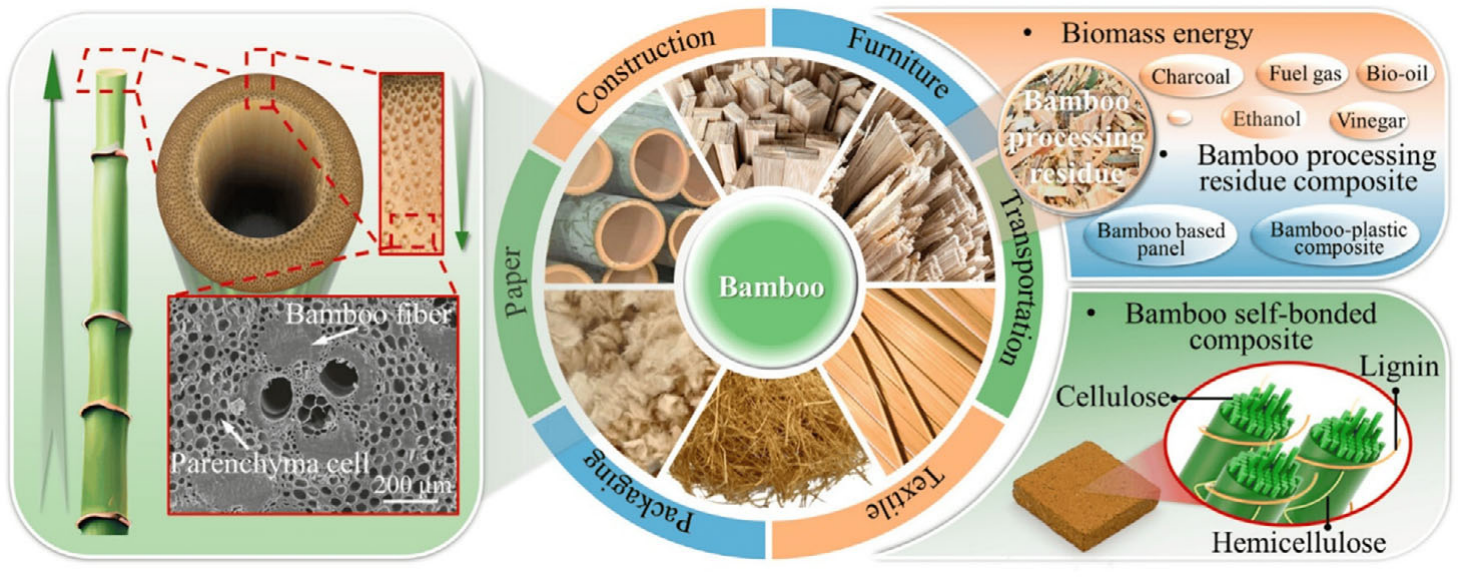
The structural characteristics and present‐day uses of bamboo. Reproduced with permission.^[^ ^140^ ^]^ Copyright 2023, American Chemical Society.

Bamboo holds significant cultural and ecological significance in numerous countries spanning Africa, America, and Asia due to its various social, ecological, and economic advantages. It offers a range of benefits, including rapid growth, independence from irrigation, elimination of the need for replanting, the ability to thrive without fertilizers, and easy harvest within a relatively short period of 3–5 years. The composition of bamboo primarily consists of lignin, cellulose, and hemicellulose.^[^ ^22^ ^]^ **Figure** **9** represents the structural properties and current applications of bamboo in recent studies. Different physical and chemical pretreatment methods have been suggested for the extraction of lignin from bamboo. The subsequent paragraph will introduce some of the latest research findings, along with the characteristics and potential applications of the extracted lignin.

An investigation to explore the efficiency of extracting lignin–carbohydrate complexes from Moso bamboo obtained from Jiangxi Province, China. The extraction process involved utilizing formic acid in aqueous solutions with varying concentrations and reaction temperatures under normal pressure to determine the optimal conditions for lignin extraction. The bamboo was ground into a powder and extracted using a Soxhlet extractor. The powder was mostly cellulose (46.80%), hemicellulose (26.03%), and lignin (27.83%). Dried bamboo powder and formic acid aqueous solution at various concentrations were mixed. The reaction mixture was filtered and concentrated by evaporation. After adding acetone to the concentrated solution. The maximum lignin yield of 24.47% was achieved with a 90% formic acid aqueous solution at 100°C for 2 h. NMR study showed that all extracted lignin samples were homogeneous, indicating that their structure remained unaltered. As shown by RSI values of 0.47 to 1.28, lignin–carbohydrate complex (LCC) samples have substantial antioxidant ability. This study opens up new uses for lignocellulose's easily degradable component.^[^ ^84^ ^]^ Maleic acid (MA) was also evaluated as a mild lignin extractor using an acidic pretreatment method followed by organic solvent fractionation to improve the valorization of lignin. The study used dried bamboo powder from HYA Group Co. Ltd. from Fujian, China, which included 44.89% cellulose, 18.37% hemicellulose, and 26.95% lignin. A 55% MA solution was added to bamboo biomass with a 1:15 loading at 120°C temperature for 1 h reaction for the mixture. The mixture was then filtered to produce lignin‐rich filtrate. The filtrate was diluted twice with deionized water to precipitate lignin, centrifugation separated maleic acid‐extracted lignin (MAeL) for organic solvent fractionation. MAeL dissolved slowly in ethyl acetate, ethanol, acetone, and dioxane/water. After each dissolution step, centrifugation separated the liquid supernatant, and rotary evaporation recovered the lignin fraction. The insoluble substrate was reacted for 2 h and precipitated as lignin in the next organic solvent. After fractionation, the lignin fractions were named MAeL‐P, MAeL‐1, MAeL‐2, MAeL‐3, and MAeL‐4. Drying the parent MAeL and lignin fractions helped characterize them. The dispersity index (Đ) of lignin decreased from 2.86 to 1.25 after organic solvent fractionation, showing increased purity and homogeneity. The lignin extracted with ethyl acetate has the highest phenolic hydroxyl (2.45 mmol g^−1^) and lowest aliphatic—OH (0.65 mmol g^−1^). Using ethyl acetate led to the recovery of hydrophobic substrates with C–C links and partial β—*O*—4 bond cleavage. Lignin fragments with varied primary structures and functional groups were obtained through successive fractionation, suggesting interesting uses and extensive possibilities for lignin preparation.^[^ ^85^ ^]^ Lignin extraction employing alkali and IL pretreatment for bamboo obtained from a bamboo‐processing facility in Seri Iskandar, Perak, Malaysia, was studied to investigate the characteristics of extracted lignin. Initially, bamboo biomass was pretreated with NaOH at 125 °C. Diluting the dark solution with ultrapure water separated cellulose‐rich materials. The solid component was filtered, washed with ultrapure water, and dried in an oven at 80 °C for 24 h. During the IL pretreatment, biomass was immersed in BmimCl and heated. To precipitate, the very viscous mixture was swiftly added to acetone and water under strong agitation. The precipitates were filtered, and acetone was extracted from the IL pretreatment filtrate under reduced pressure, precipitating lignin. Both filtrates were acidified for lignin precipitation. After centrifugation, precipitates were filtered, washed with pH 2.0 water, and dried in an oven before analysis. The results showed that pretreatment strategies affected extracted lignin characteristics. Alkali and IL treatments removed 42% and 11% of acid‐insoluble lignin. Due to insufficient hemicellulose removal, alkaline treatments yielded more. Analysis showed that Bmim Cl IL pretreatment can extract pure lignin without additional purification procedures, making it better than alkali pretreatment. IL‐extracted lignin was also more thermally stable than alkali‐extracted lignin.^[^ ^86^ ^]^ Using ultrasound‐assisted organosolv pretreatment, the research aimed to recover high‐quality lignin and cellulose‐rich pulp from bamboo. The study evaluated ultrasonic and organosolv pretreatment on bamboo from IIT Guwahati, Assam, India, using RSM. The bamboo was mixed with acidic aqueous ethanol in an ultrasonic bath and an autoclave digester. Optimization was conducted using RSM with a 3^3^‐CCD factorial design to assess the impact of pretreatment temperature (140–180 °C), duration (30–90 min), and ultrasound time (15–45 min) to maximize lignin yield. The optimum conditions were 180 °C, 55 min pretreatment, and 30 min ultrasound, yielding 65.81 ± 2.40% lignin with 95.37 ± 1.17% purity. Comparing lignin yield and delignification with and without sonication on organosolv pretreatment showed its effect. The lignin yield with sonication in optimum conditions yielded 65.81 ± 2.40%, which was higher than the nonsonicated procedure (57.96 ± 3.76%). The primary variable influencing lignin yield was temperature. Lignin–carbohydrate linkages were cleaved by ultrasound, improving delignification and cellulose crystallinity. Sonication enhanced lignin quality, as shown by NMR, FTIR, GPC, and thermogravimetric analysis (TGA). Ultrasound pretreatment employs ultrasonic waves to generate cavitation, which in turn creates shear forces that disturb the complex structure of LCB. This disruption enhances the fractionation of the main components of LCB, including lignin.^[^ ^87^ ^]^ Extracted lignin has many β—*O*—4 bonds, making it suitable for future applications.^[^ ^88^ ^]^ A study examined the lignin characteristics of bamboo from forests in Kamahi Devi, Hoshiarpur District, using various pretreatment methods, including acid treatment, alkali treatment, and sequential organosolv treatment. The acid extraction process comprised combining bamboo fibers with sulfuric acid in a beaker in a 20 °C bath for 2 h. The mixture was then diluted with 3% H_2_SO_4_. Boiling the solution for 4 h with hot water while maintaining the solution volume, then filtering and rinsing with hot water after settling the insoluble material overnight. The precipitates were oven‐dried. In alkali extraction, bamboo fibers were treated with hot water, separated, and pulped with sodium hydroxide, fiber filtration generated black liquor, which was acidified to pH 2 with 5 N sulfuric acid. Lignin precipitated during acidification was washed and dried in ambient air. For organosolv, bamboo fibers were treated with formic and acetic acids. FA/AA‐treated pulped fibers were delignified in a hot water bath with peroxyformic and peroxyacetic acids. After delignification, fibers were filtered and washed with hot water. The delignified fibers were bleached with H_2_O_2_. A final washing with distilled water removed any leftover lignin from the pulp. Adding five times more distilled water than the concentrated liquor precipitated lignin in formic acid. The precipitated lignin was Buchner funnel‐filtered, washed with distilled water, and dried, then all the extracted lignin from different procedures was characterized. The results illustrated that organosolv pretreatment yielded the most lignin from bamboo biomass. The solvent actions of formic acid/acetic acid (FA/AA) and the oxidizing effects of peroxyacid helped dissolve lignin in a hydrogen peroxide medium. The results also showed that the extraction process affected lignin thermal characteristics. Acid‐extracted lignin was most thermally stable and pure.^[^ ^89^ ^]^ A pretreatment method involving a hydrated DES was investigated to obtain light‐colored lignin from bamboo shoot shells (BSS) obtained from a food factory in Sichuan province, China. The hydrated DES consisted of formic acid, benzyl triethylammonium chloride (BTEAC), and varying water contents. The DES formulations with different water contents were labeled as H0 (pure DES), H10 (DES with 10% water content), H30 (DES with 30% water content), and H50 (DES with 50% water content). To prepare the DES, formic acid and BTEAC were mixed in a 1:1 ratio (w/w) under stirring and heating (80 °C) for 2 h. Subsequently, the hydrated DES was obtained by mixing the DES with water for 1 h under the same conditions. The filtrate obtained from solid–liquid separation was concentrated using rotary evaporation. To recover the lignin, deionized water was added to the concentrate, and the collected lignin samples underwent three additional rounds of washing with water to remove impurities. It was found that under optimized conditions, the pretreatment method resulted in 82.9% delignification and 83.7% lignin recovery, with 87.4% preservation of the β‐*O*‐4 linkages in the obtained lignin sample labeled as L‐H30. Notably, L‐H30 exhibited significant antioxidant ability, tyrosinase inhibitory capacity, and sun protection properties. When 5% of L‐H30 was added to a sun cream formulation, it led to a 120% increase in the SPF value (SPF = 32.6) compared to the baseline SPF of 14.8. The extraction of light‐colored lignin using the hydrated DES demonstrates promising opportunities for the multifunctional utilization of lignin in cosmetic applications.^[^ ^90^ ^]^

In conclusion, various pretreatment methods for lignin extraction from bamboo demonstrated different degrees of extraction. The highest lignin recovery was 83.7% achieved using hydrated DES pretreatment, and ultrasound‐assisted organosolv pretreatment achieved 65.81%, with corresponding purity levels of 87.4% and 95.37%, respectively. IL pretreatment extracted highly pure lignin but with a lower yield of 11%, while alkali treatment achieved a higher lignin removal rate of 42% but with reduced purity. Hydrated DES pretreatment, utilizing formic acid and benzyl triethylammonium chloride (BTEAC) with varying water contents, can be considered the most effective method due to its high delignification efficiency, lignin recovery, and β‐*O*‐4 linkage preservation. Further enhancements could involve integrating ultrasound or microwave assistance, which has been shown to improve outcomes, particularly in organosolv pretreatment. For example, lignin yield with sonication under optimal conditions reached 65.81%, significantly higher than the yield from the nonsonicated procedure, 57.96%. **Table** **5** shows a summary of the literature on lignin extraction, charactarization and applications from bamboo waste.

**Table 5:** Overview of studies for the lignin extraction from bamboo waste.

Biomass	Method	Further info	Yield	Properties	Prospect/application	Ref.
Moso bamboo	Acidic pretreatment	Formic acid aqueous solution concentrations (70 % to 100 %) and reaction temperatures (40 to 100 °C) under normal pressure	24.47%	DTGmax of 354 °C was achieved at 100 °C, demonstrating good thermal stability. DPPH free radical scavenging index increased from 0.51 to 1.28 as extraction temperature rose from 40 to 100 °C, indicating higher antioxidant activity.	Antioxidant	[84]
Dried bamboo powder	Acidic pretreatment	55% MA maleic acid solution followed by organic solvent fractionation	–a)	The phenolic hydroxyl content of lignin extracted by ethyl acetate was the highest (2.45 mmol/g), and the aliphatic‐OH concentration was the lowest (0.65 mmol/g). The glass transition temperature after lignin fractionation was lower than that of raw lignin (122–150 °C vs. 159 °C),	Epoxy resin, rubber, thermoplastic additives, and polymer raw materials	[85]
Bamboo “buluh Semantan”	Alkali and ionic liquid pretreatment.	Alkali pretreatment utilizing a NaOH solution and ionic liquid (IL) pretreatment using BmimCl	42% 11%	The degradation onset temperature of alkali and ionic liquid extracted lignin were found to be 262.20 and 172.41 ºC, indicating that IL extracted lignin has higher thermal stability compared to alkali extracted lignin.	Aldehydes, phenol, hydrocarbons, and aromatics	[86]
Bamboo “Bambusa tulda”	Organosolv pretreatment	Ultrasound‐assisted acidic aqueous ethanol	65.81 ± 2.40%	Ultrasonicated Organosolv US‐OS lignin has a lower carbon‐carbon (β‐β) linkage (17.31%) compared to nonsonicated (25.73%), indicating the effect of sono‐assisted OS pretreatment in producing high‐quality lignin. The US‐OS method yielded 65.81 ± 2.40% lignin, compared to 57.96 ± 3.76% for the nonsonicated process	–a)	[88]
Bamboo Bambusa polymorpha	Acid treatment (klason method), alkali treatment and sequential Organosolv treatment	Acid treatment using 72% sulphuric acid Alkali treatment using 15% sodium hydroxide Organosolv treatment using formic acid/acetic acid/peroxyformic acid/peroxyacetic acid/H_2_O_2_.	14.6% 9.2% 22.6%	The highest yield was 22.6% from organosolv. bamboo has unique HGS lignin composed of *p*‐hydroxyl phenylpropane (identified at the bands appear between 1600 and 1500 cm_1. The bands near 1300 cm_1 and 1200 cm_1 indicate syringyl (S) and guaiacyl (G), with a high S/G ratio of 1.1. DTG_max_ was between 320 and 340 °C for all lignin samples. Klason lignin exhibits the strongest thermal stability.	–a)	[89]
Bamboo shoot shells (BSS)	Hydrated deep eutectic solvent (DES)	The hydrated DES consisted of formic acid, benzyl triethylammonium chloride (BTEAC), and varying water contents	–a)	Increased antioxidant activity as DES extracted lignin surpasses BHT used as reference (L‐30, RSI = 3.9) (BHT, RSI = 2.2). Lignin samples at low conc had higher tyrosinase inhibitory capacity (27.3%‐41.5%) better than pHB (16.2%). SPF value increased from 14.8 to 32.6 compared to commercial lignin.	Sun cream	[90]

a) Indicates information was not reported in cited literature.

### 3.4 Extraction of Lignin from Rice Waste

**Figure 10:**
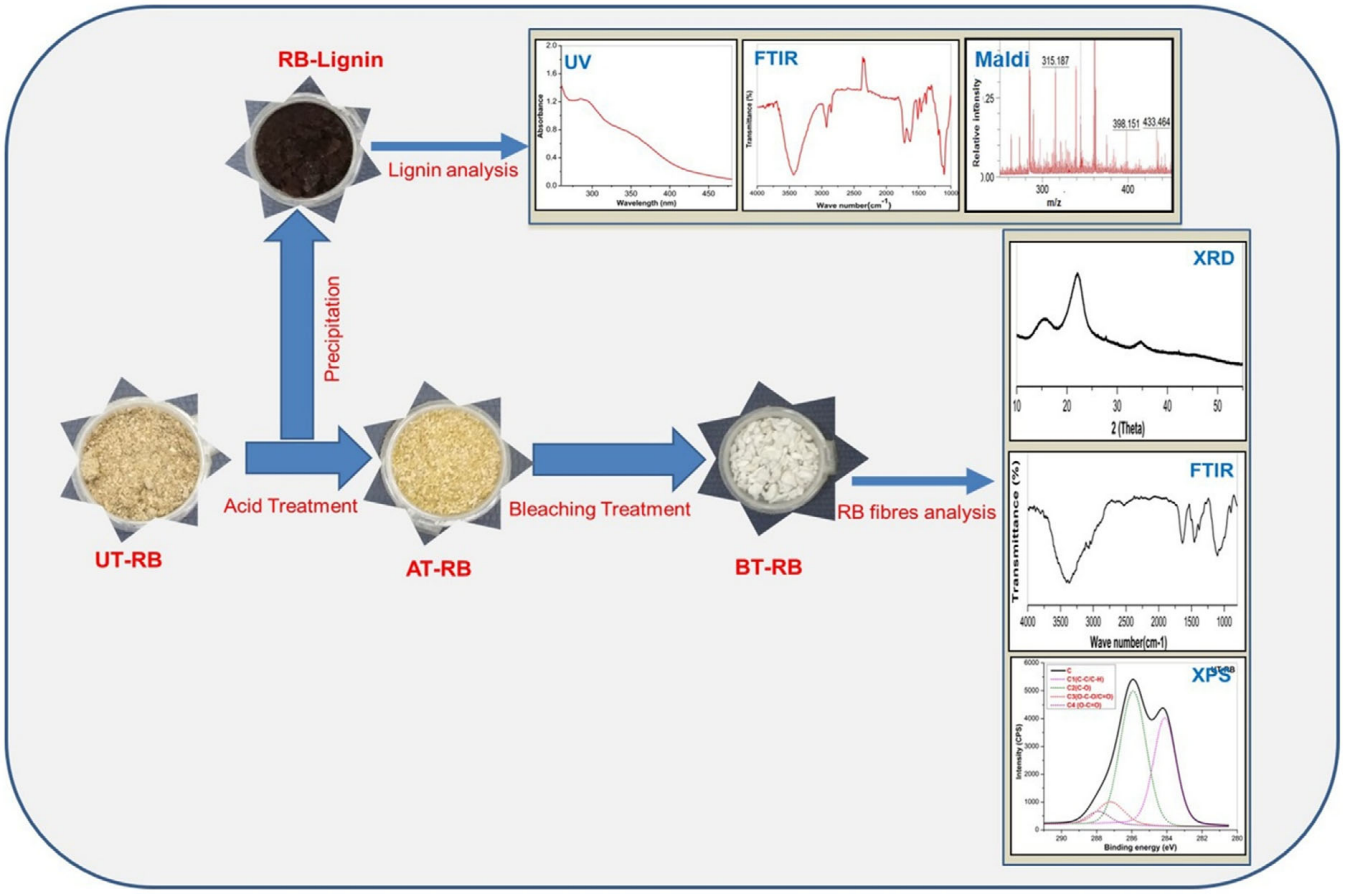
Schematic representation of lignin and cellulose fractionation steps and characterization from rice bran. Reproduced with permission.^[^ ^94^ ^]^ Copyright 2020, Elsevier.

Many people, especially in Asia and Africa, rely on rice as their primary staple food. However, rice production annually generates significant trash, including 8 × 10^11^ kg of straw and 1.5 × 10^11^ kg of husks.^[^ ^91^ ^]^ Only 20% of rice straw is used to make biofuels, paper, fertilizers, and animal feed.^[^ ^92^ ^]^ The rest of the straw is burned or utilized as crop mulch after harvest. Meanwhile, burning is becoming socially unacceptable due to air pollution in the surrounding environment. This waste material includes 7%–10% lignin and 30%–35% cellulose, making it a cost‐effective feedstock for lignin and cellulose synthesis.^[^ ^93^ ^]^ **Table** **6** shows a summary of the literature on lignin extraction from rice waste. Rice bran serves as a valuable source of lignin and cellulose. Following the pretreatment process, which effectively separates lignin and cellulose fibers from rice bran, the extracted lignin and cellulose are thoroughly characterized using advanced analytical techniques such as FTIR, X‐ray photoelectron spectroscopy, scanning electron microscopy (SEM), X‐ray diffraction (XRD), and additional FTIR analysis, as illustrated in **Figure** **10**. These analyses confirm the suitability of the extracted lignin and cellulose as feedstocks for integrated biorefineries. Furthermore, their properties position them as sustainable alternatives to petroleum‐derived resources, supporting the development of eco‐friendly and renewable materials.^[^ ^94^ ^]^

**Table 6:** Overview of studies for the lignin extraction from rice waste.

Biomass	Method	Procedure	Yield	Ref.
Rice bran	Acidic pretreatment	Organic acid solution (70 parts formic acid and 30 parts acetic acid) bleaching treatment, an aqueous sodium chlorite Acidification using acetic acid	–a)	[94]
Rice straw	Alkaline pretreatment	Sodium hydroxide (NaOH) 5 wt% with ethylene glycol solution as a co‐solvent.	lignin recovery 94% b)	[95]
Vietnam's rice straw	Ultrasound‐Assisted Alkaline Treatment	Treatment with 2 M NaOH, then lignin precipitation with HCl to pH 5.5	84.7%	[96]
Rice straw	Acidic and alkaline pretreatment	alkaline treatment utilizing sodium hydroxide organic acid treatment employing formic acid and acetic acid mixture	–a)	[145]
Rice husks (Oryza sativa, Asian rice, RHs)	Organosolv	H_2_SO_4_ solution with water‐ethanol mixture	–a)	[97]
Asian rice husks from India	Ionic Liquid	BAILs including [BMIM]Cl [C_3_SO_3_HMIM] Cl [C_3_SO_3_HMIM] [Ace] [C_3_SO_3_HMIM] [HSO_4_] [C_4_SO_3_HMIM] Cl [C_4_SO_3_HMIM] [Ace] [C_4_SO_3_HMIM] [HSO_4_] dissolved rice husk then acetone and water to precipitate cellulose‐rich compounds, then to produce extract lignin the solution was vacuum‐filtered.	–a)	[98]
Rice husk from India	Deep eutectic solvents	RH was mixed choline chloride/pTSA and the reaction was carried out at variable water content reaction temperature and reaction time, then water was used as anantisolvent to precipitate solubilized lignin. Then by filtration	87.8%	[99]

a) Indicates information was not reported in cited literature.

b) literature reported lignin recovery instead of lignin yield.

The recovery of high‐purity lignin from rice straw provided by Far Eastern New Century Co., Taiwan's. Alkaline soda pretreatment with ethylene glycol co‐solvent treated rice straw waste. The improved reaction time of 300 min at 190 °C yielded 94% lignin recovery and 92% purity. Ethylene glycol increased lignin solubility, increasing yield and recovery compared to the soda method without a cosolvent. A kinetic study was done. Analysis showed that the soda‐ethylene glycol delignification reaction has two phases, a quick bulk phase and a slow residue phase in the process. At least 80% of the lignin was rapidly solubilized in the fast phase due to nucleophile attacks on its ether bonds. The slow phase degraded C—C linkages in the residual lignin.^[^ ^95^ ^]^ To improve economic efficiency and reduce environmental pollution. Treatment of the rice straw with ultrasonic irradiation. Pretreatment with NaOH removed cellulose from biomass. The acidified hydrolysate was precipitated in 95% ethanol to isolate hemicellulose. Precipitation of alkali‐soluble lignin at pH 1.5, regulated by HCl, yielded lignin. With ultrasonic irradiation, the extraction time was lowered from 2.5 h to 1.5 h for the same yields. Moreover, for 30 min, lignin separation yields rose from 72.8% to 84.7%. Ultrasonic‐assisted alkaline treatment produced high‐purity, high‐molecular‐weight lignin from rice straw. Lignin and cellulose derived from rice straw had superior thermal stability, with just 5% degradation at temperatures exceeding 230 °C.^[^ ^96^ ^]^ To delve deeper into the differentiation between acidic and alkaline pretreatment, researchers conducted an analysis of lignin extraction and characterization from waste rice straw sourced from fields in the vicinity of Chennai, India. Two treatments were used: alkaline sodium hydroxide and organic acid, a formic acid/acetic acid mixture. Lignin fractions from alkaline and organic acid treatments were called SHT‐lignin and OAT‐lignin, respectively. To determine the phenolic content and antioxidant capabilities of the isolated lignin fractions, UV–vis, FTIR, and 1^ H^ NMR were used for structural analysis. The results showed that SHT‐lignin has 28.87 mg GAE/g total phenolic content and 59.50% DPPH radical scavenging activity. DPPH radical scavenging activity was 45.74%, and total phenolic content was 24.75 mg GAE/g in OAT‐lignin. SHT's basic conditions solubilize phenolic hydroxyl groups better than OAT's acidic conditions, explaining this disparity. The atomic O/C ratio values of untreated and treated biomass showed selective lignin removal in the SHT process, while the OAT process fractionated lignin and hemicelluloses completely.^[^ ^94^ ^]^

Asian rice husks labeled RH I, RH II, and RH III from various Indian locations were organosolv pretreated. The goal was to compare the physical and chemical properties of lignin from several rice husk types. Organosolv lignins (ORGLs) were isolated using water‐ethanol and H_2_SO_4_ at 180 °C for 1 h. The isolated lignins’ structural properties were analyzed using advanced characterization methods. XRD showed no polysaccharide contamination in isolated lignin samples. XRD, GPC, and elemental analysis showed that lignins from diverse rice husk kinds have similar bulk‐level characteristics; meanwhile, despite the same isolation technique, molecular‐level examination employing UV–vis, ATR, and NMR revealed differences in lignin sample substructure concentrations (G, H, S, and T). Certain aromatic rings lacked substituents due to double bond equivalency. Interestingly, RH III‐derived lignin has a larger concentration of guaiacyl (G) units and (T) Tricin subunits than RH I and RH II‐derived lignins.^[^ ^97^ ^]^ The potential of Bronsted acidic ionic liquids (BAILs) as green solvents for lignin dissolution and depolymerization was evaluated, where a series of BAILs were synthesized, characterized, and employed as medium solvents to fractionate rice husk from BERNAS rice mill, Selangor, Malaysia, into lignin, cellulose, and hemicellulose. [C_3_SO_3_HMIM] [Cl] and [Ace] removed 78% and 53% of regenerated lignin from rice husk, respectively, while [HSO_4_] extracted just 11%. The regenerated lignin and cellulose were FTIR and TGA‐characterized. BAIL [C_3_SO_3_HMIM] Cl was an excellent medium solvent for rice husk lignin extraction and could be recycled four times. Further depolymerization of regenerated lignin demonstrated that [C_3_SO_3_HMIM] [HSO_4_] is a good solvent for low‐molecular‐weight aromatic compounds. Recyclability, thermal and chemical stability, and energy savings are benefits of using BAILs as lignin extraction and depolymerization solvents.^[^ ^98^ ^]^

DESs are prepared through a simple synthesis process that utilizes readily available, nontoxic, and cost‐effective materials, making them a more practical alternative to ILs. In the context of lignin extraction from rice husk sourced from a local vendor in Thanjavur, Tamil Nadu (India), a DES comprising choline chloride and *p*‐toluene sulfonic acid (pTSA) as a biodegradable hydrogen bond donor was employed. This DES demonstrated effective lignin extraction with uniformity. Under optimized reaction conditions, an 87.8% lignin yield was attained. Notably, the phenolic content reached 818 and 622 μg FAE/mg at 110 °C and 180 min, respectively, which significantly exceeded the phenolic content of commercial lignin (519.5 μg FAE/mg). The impact of reaction temperature on phenolic content outweighed that of reaction time. Phenolic compounds, indicative of antioxidant potential, contributed to the observed good antioxidant activity in the extracted DES‐lignin, as these compounds can function as reducing agents.^[^ ^99^ ^]^

In summary, among the discussed various pretreatment methods for extracting lignin from rice waste, the highest lignin yield, 94%, and purity of 92% were achieved using soda‐ethylene glycol as a co‐solvent with NaOH alkaline pretreatment at 190 °C for 300 min. The addition of ethylene glycol improved solubility and accelerated delignification compared to traditional soda methods. DESs also demonstrated effective lignin extraction, yielding 87.8% lignin with superior phenolic content (818 μg FAE/mg), which indicates its suitability for antioxidant applications.

### 3.5 Extraction of Lignin from other Biomasses

While this review initially concentrated on palm tree waste, bagasse, bamboo, and rice waste—biomass sources with substantial research data to facilitate comparative analysis—it is equally critical to address other underutilized but promising feedstocks such as corn stalks, cotton stalks, and wheat straw. These agricultural residues are not only abundant but also exhibit diverse chemical compositions, making them valuable candidates for lignin extraction and valorization. This subsection provides a comprehensive analysis of lignin extraction methodologies tailored to these biomass types while addressing the recent advances in lignocellulosic pretreatment techniques and evaluating their efficiency, scalability, and potential for industrial applications. By expanding the scope to include these additional feedstocks, we aim to underscore the versatility and adaptability of lignin extraction technologies, demonstrating their broader applicability across various lignocellulosic resources.

Switchgrass (*Panicum virgatum* L.), sourced from the University of Tennessee‐Knoxville's experiment fields at the Plant Sciences Unit of the East Tennessee Research and Education Center (ETREC), was pretreated with Cyrene, a sustainable biomass‐derived solvent. Cyrene, also known as bio‐based dihydrolevoglucosenone, offers a green and renewable alternative to traditional petroleum‐based dipolar aprotic solvents. It is synthesized from cellulose in a straightforward two‐step process and is reported to have significant potential in biomass processing.^[^ ^100^ ^]^

The extraction of lignin from the extractive‐free switchgrass involved using a Cyrene/water mixture with Cellulolytic enzyme lignin (CEL) was also isolated from untreated switchgrass through enzymatic hydrolysis with Accellerase 1500 in sodium acetate buffer, followed by extraction with a 1,4‐dioxane/water mixture and recovery via rotary evaporation and freeze‐drying. Cyrene pretreatment efficiency improved with prolonged reaction time and higher catalyst concentration. Delignification reached 61.8% after 1 h pretreatment using a Cyrene/water 2:1 mixture with 33 mM H_2_SO_4_, while increasing the acid concentration to 67 mM further improved delignification to 73.3%. Sulfuric acid significantly enhanced delignification, reducing lignin molecular weight by 20%–60% compared to CEL while maintaining similar molecular weight dispersity (Đ = 2.67 ± 0.3 *vs*. CEL Đ = 2.64). High‐resolution 2D HSQC NMR analysis confirmed Cyrene's ability to preserve lignin's structural integrity, particularly β‐*O*‐4 linkages, while simultaneously reducing aliphatic hydroxyl groups and increasing phenolic hydroxyl content by ≈10%–12%. These structural modifications are favorable for the downstream application of lignin, underscoring the promise of Cyrene as an effective, sustainable solvent for biomass pretreatment.^[^ ^101^ ^]^

Scotch pine (*Pinus sylvestris* L.), a tree species from the family Pinaceae and native to Eurasia, plays a significant role in forest management across many European countries and is considered a biomass with a high lignin content.^[^ ^102^ ^]^ Zheng et al. studied scotch pine sourced from Jiangsu, China, with a lignin content of 28.60 ± 0.13%, to investigate the pretreatment of LCB using a Lewis acid‐based deep DES. The isolated lignin's color and sun protection properties were evaluated using a Datacolor 200 spectrophotometer, demonstrating the potential of light‐colored lignin for UV protection applications. The DES was synthesized by combining choline chloride (ChCl), various polyols (1,4‐butanediol, glycerin, propylene glycol, or ethylene glycol), and a Lewis acid (ZnCl_2_, AlCl_3_, CuCl_2_, or FeCl_3_). The results showed that the different polyols served as HBD in the DES, resulting in lignin yield following the order PG > EG > BDO > Gly. These results reflect the effect of the hydrogen bonding strength and interactions between DES components and lignin, which play a critical role in the dissolution process. Prolonging the pretreatment time from 0.5 to 3.0 h significantly increased lignin yield, from 31.7 ± 3.5% to 59.1 ± 1.3%, due to the progressive breakdown of lignin–carbohydrate bonds. Among the DES systems tested, the ternary DES comprising ChCl, 1,4‐butanediol, and AlCl_3_ produced lignin with the lightest coloration (brightness value, L* = 77.9), a yield of 32.2%, and minimal condensation. Characterization using 2D‐HSQC and ^3^ ^1^P NMR revealed a high retention of β‐*O*‐4 linkages (95.8%) and an increase in phenolic hydroxyl groups, enhancing its suitability for UV absorption applications. Enzymatic hydrolysis and UV transmittance experiments confirmed its potential for high‐value applications, including sunscreens, achieving an SPF of 26.95 with 5% lignin incorporation. These results highlight the effectiveness of AlCl_3_‐based DES in producing high‐quality lignin for industrial applications, particularly in the development of eco‐friendly UV protection products.^[^ ^103^ ^]^

Poplar sawdust, sourced from Xuzhou, Jiangsu Province, China, was utilized to evaluate and compare acid‐ and alkali‐catalyzed 1,4‐butanediol (BDO) organosolv pretreatments for the co‐production of fermentable sugars and high‐value lignin. Acid‐catalyzed BDO pretreatment was conducted using aqueous solution (BDO‐water ratio of 65:35, v/v) at 170 °C for 60 min using HCl at a solid‐to‐liquid ratio of 1:7, while alkali‐catalyzed pretreatment under the same conditions employed NaOH. Following pretreatment, the solid fraction was separated, washed, and stored, while lignin was recovered from the liquid fraction via direct precipitation (acid‐BDO) or pH adjustment with H_2_SO_4_ (alkali‐BDO), followed by centrifugation, rinsing, and freeze‐drying.

The acid‐catalyzed BDO pretreatment demonstrated superior performance, removing up to 82.04% of lignin and achieving a maximum sugar yield of 79.41%, surpassing alkali‐catalyzed pretreatment in both lignin removal and enzymatic digestibility. Characterization of lignin revealed that acid‐BDO lignin retained a higher molecular weight (4032 g mol^−1^) compared to alkali‐BDO lignin (2434 g mol^−1^), attributed to limited depolymerization and controlled polymerization. FTIR and ^3^ ^1^P NMR analyzes indicated a significant increase in phenolic hydroxyl (OH) content during acid pretreatment (0.60 mmol g^−1^) compared to alkali pretreatment (0.50 mmol g^−1^), resulting from β‐*O*‐4 linkage cleavage and enhanced hydrophilicity.

The enhanced phenolic hydroxyl formation and reduced lignin repolymerization observed during acid pretreatment improved enzymatic hydrolysis. Additionally, lignin derived from acid‐BDO pretreatment exhibited superior radical scavenging activity (RSI = 0.76), attributed to its higher phenolic OH content and optimal molecular weight. These findings highlight the potential of acid‐catalyzed BDO organosolv pretreatment as an effective strategy to produce value‐added lignin with enhanced functional properties.^[^ ^104^ ^]^

A novel pretreatment technique was investigated using the Hybrid poplar line NM6 *(Populus nigra and Populus maximowiczii),* sourced from the Great Lakes Bioenergy Research Center (GLBRC), to examine lignin structural changes during extraction with γ‐valerolactone (GVL) under varying conditions. Two hydrolysis methods were compared: Single‐Step Hydrolysis: Conducted at 80–120 °C for 1–24 h, using a solution of 85 mM sulfuric acid in a 9:1 GVL‐to‐water ratio, with a 10% solid content. Two‐Step Hydrolysis: Involved two consecutive treatments with acidic GVL‐water solution at 90 °C for 1.5 h per step. After the first step, the biomass was treated with fresh acidic GVL, and the lignin‐rich solution was separated by filtration. Lignin was precipitated by diluting the solution with water, followed by centrifugation, rinsing, and vacuum‐drying at 50 °C, yielding two lignin samples.

The two‐step hydrolysis demonstrated superior performance, achieving a higher lignin yield (56.5%) compared to the single‐step process (54.8%). FTIR analysis revealed that the two‐step process preserved significantly more β‐*O*‐4 linkages (31.9% vs. 10.6%), a critical indicator of lignin quality. Further characterization using 2D HSQC NMR, ^31^ P NMR, and GPC showed that increasing temperature and time during pretreatment enhanced lignin yield but led to condensation, reduced aliphatic hydroxyl content, and increased molecular weight. The multi‐step process minimized lignin degradation, retaining three times more β—*O*—4 bonds than the single‐step method. These findings underscore the potential of GVL as an effective solvent for extracting high‐quality lignin that is suitable for advanced applications. The study also highlights the scalability of the process using continuous flow systems for industrial applications.^[^ ^105^ ^]^

Cotton, accounting for 40% of global fiber production, yielded 26.9 million metric tons (MMT) in 2018. Cotton stalks, an abundant agricultural residue generating ≈50 million tons annually (2 tons per hectare in India), represent a low‐cost carbon source for producing value‐added bioproducts like fuel ethanol.^[^ ^106^ ^]^ Cotton stalks from Kashgar, Xinjiang, were used to compare two lignin extraction methods—ionic liquid (ILL) and ultrasonic‐assisted ionic liquid (UILL)—while optimizing pretreatment conditions and assessing lignin's antioxidant efficiency in polypropylene (PP) synthesis.

Four ILs were evaluated for ILL:1‐ethyl‐3‐methylimidazolium acetate ([Emim][CH3COO]), 1‐allyl‐3‐methylimidazolium chloride ([Amim]Cl), 1‐butyl‐3‐methylimidazolium chloride ([Bmim]Cl), and 1‐ethyl‐3‐methylimidazolium chloride ([Emim]Cl). Pretreatment involved a 1:20 solid‐to‐liquid ratio, heating at 110–170 °C for 0.5–3 h. For UILL, pretreated powder was mixed with an IL–water solution and processed ultrasonically (45 kHz, 100 MPa). After treatment, lignin was isolated through filtration, centrifugation, and recovery of ILs via rotary vacuum evaporation, followed by purification and freeze‐drying. UILL demonstrated superior efficiency (94.01%) compared to ILL (93.63%), yielding lignin with enhanced thermal stability and antioxidant performance. The study revealed that 1‐ethyl‐3‐methylimidazolium acetate ([Emim][CH3COO]) exhibited the highest lignin extraction efficiency among the tested ILs, attributed to its cation‐controlled solubility and strong π—π bonding and solvent interactions with lignin. Optimal extraction conditions were reported at a temperature of 150 °C and a 1h duration, achieving a maximum lignin yield of 76%. Prolonged durations and higher temperatures reduced yields due to molecular degradation and byproduct formation. FTIR analysis revealed abundant functional groups, particularly phenolic hydroxyls, with UILL lignin exhibiting higher phenolic content, contributing to its superior antioxidant properties. TGA confirmed UILL lignin's improved thermal stability, while SEM images revealed uniform spherical particles. Incorporating UILL lignin into PP enhanced oxidative resistance (78.72% increase in oxidation induction time) and thermal stability without compromising its mechanical properties.^[^ ^107^ ^]^ These results emphasize the significance of tailoring pretreatment methods to specific biomass sources to achieve optimal lignin yield and desired properties for diverse applications. UILL achieved the highest delignification at 94.01%, followed by acid‐catalyzed BDO at 82.04%. **Table** **7** summarizes the presented research for lignin extraction from the discussed biomasses and their characterization and application. Table 7 overview of studies for the lignin extraction from diverse biomasses.

**Table 7:** Overview of studies for the lignin extraction from diverse biomasses.

Biomass	Method	Procedure	Yield	Properties	Application/ prospect	Ref
Switchgrass	Organosolv	1: 15 solid loading of switchgrass with Cyrene/water mixture with stirring for 10 min. The pretreatment was conducted in a reactor at 120 °C for 1–4 h using varying concentrations of sulfuric acid as a catalyst.	34.9	20%–60% molecular weight reduction Dispersity index (Đ) ~2.67. β‐*O*‐4 linkage preserved	–a)	[101]
Scotch pine	Deep eutectic solvent	Choline Chloride (ChCl), different alcohols (1,4‐butanediol glycerin, propylene glycol, and ethylene glycol), and 4 different Lewis acid catalysts (ZnCl_2_, AlCl_3_, CuCl_2_, or FeCl_3_) (1,4‐BDO, EG, PG, or Gly), Pretreatment conditions: solid‐to‐liquid ratio 1:15 and reacted at 80–150 °C for 0.5–3 h	32.2% (For ChCl: BDO at 100 °C and 1 h)	Lignin from AlCl_3_‐DES was the lightest among all samples (L* = 77.9). Total hydroxyl groups: 3.78 mmol/g.	Sunscreen with SPF 26.95	[103]
Poplar sawdust	Organosolv	pretreatments of poplar biomass with an aqueous solution (BDO‐water ratio of 65:35, v/v) at 170 °C for 60 min with HCl for Acid‐catalyzed BDO and NaOH for alkali‐catalyzed BDO pretreatment and a solid‐to‐liquid ratio of 1:7.	79.41% for Acid‐Catalyzed BDO	Phenolic OH Content 0.60 mmol/g Molecular Weight 4032 g/mol Antioxidant Activity 0.76 (RSI)	antioxidant	[104]
Hybrid poplar line NM6	Organosolv	Single‐step hydrolysis: a solution of 85 mM sulfuric acid in a 9:1 GVL‐to‐water ratio, with a 10% solid content. Two‐step hydrolysis where the pretreated biomass from first hydrolysis is pretreated again with fresh acidic GVL	56.5% (Two‐Step hydrolysis) 54.8% (Single‐Step hydrolysis)	Phenolic Hydroxyl Groups 3.58 mmol/g Molecular Weight 2.13 kDa	–a)	[105]
Cotton stalks	Ionic liquid – Ultrasound‐assisted ionic liquid	Pretreatment using a 1:20 solid‐to‐liquid at 110–170 °C for 0.5–3 h using ([Emim][CH3COO]), ([Amim]Cl), ([Bmim]Cl), and ([Emim]Cl). Ultrasonic processing frequency 45 kHz.	Extraction Efficiency (%) for ILL Method 93.63% and UILL Method 94.01%b)	UILL lignin had a higher decomposition temperature (250 °C) than ill (230 °C), indicating better thermal resistance.	polypropylene (PP)	[107]

a) Indicates information was not reported in cited literature.

b) literature reported lignin recovery instead of lignin yield.

In conclusion, the review highlights distinct trends and outcomes for lignin extraction from various LCB sources. Alkaline pretreatment of SCB achieved a lower lignin yield of 13%, while organosolv pretreatment of the same biomass resulted in a significantly higher recovery of 87%, along with superior thermal stability (DTG_max_ of 437 °C). Notably, the highest lignin yield was achieved through alkaline soda pretreatment with ethylene glycol as a co‐solvent for rice straw, yielding 94% lignin recovery with 92% purity, surpassing other biomass sources. Additionally, green solvents, ranging from sustainable solvents like Cyrene and γ‐valerolactone (GVL) to ILs and DESs, have been found to result in better purity levels of extracted lignin. DESs demonstrated exceptional lignin purity, such as the hydrated DES applied to bamboo shoot shells, which achieved 87.4% purity and 83.7% recovery, along with a high β‐*O*‐4 linkage preservation rate (87.4%). This improves the quality of lignin for a wide range of applications and highlights the significant progress toward efficient and environmentally friendly lignin recovery processes. Lignin characteristics varied significantly based on both biomass type and extraction method. Despite using the same IL extraction, lignins from different oil palm biomass sources exhibited distinct thermal degradation behaviors, with OPT, OPF, and OPEFB degrading at 215 °C, 207.5 °C, and 272 °C.^[^ ^66^ ^]^ Similarly, acetosolv and kraft extraction from SCB produced lignins with contrasting properties, where acetosolv lignin exhibited higher thermal stability, lower molar mass, which made it more suitable for Phenol‐formaldehyde resin.^[^ ^78^ ^]^ These results highlight the wide range in lignin characteristics even when extracted from different parts of the same biomass using the same method, therefore emphasizing the significant role of biomass composition and extraction process in establishing lignin characteristics. Further research is required to enhance the efficiency of extraction techniques while also addressing the potential difficulties that may come when implementing these methods on a larger, industrial scale.

## 4 Research and Industrial Advancements in Extracted Lignin Applications

### 4.1 Applications for Extracted Lignin

**Figure 11:**
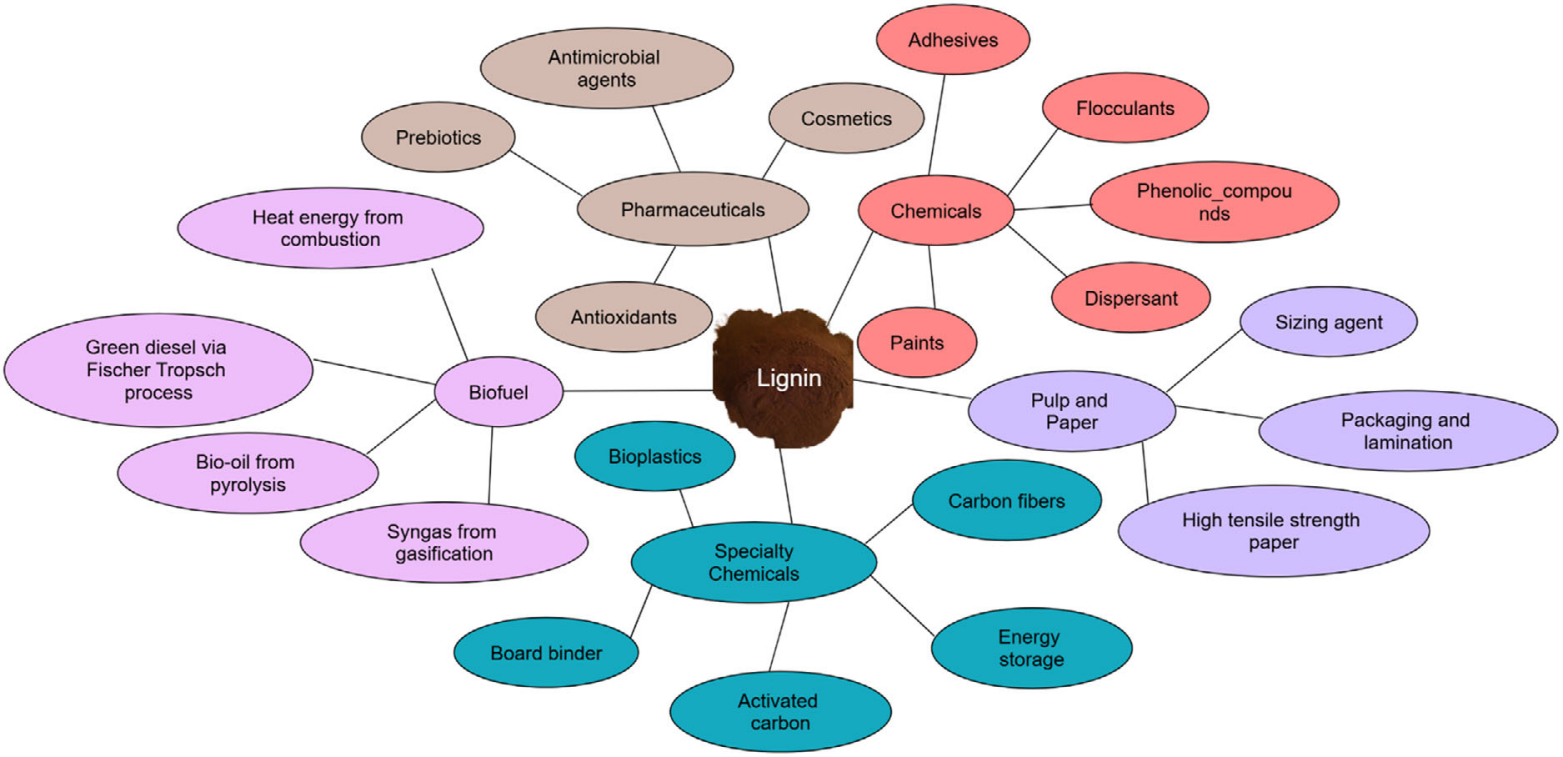
The potential applications of lignin derived from biomass. Reproduced with permission.^[^ ^26^ ^]^ Copyright 2021, Elsevier.

**Figure 12:**
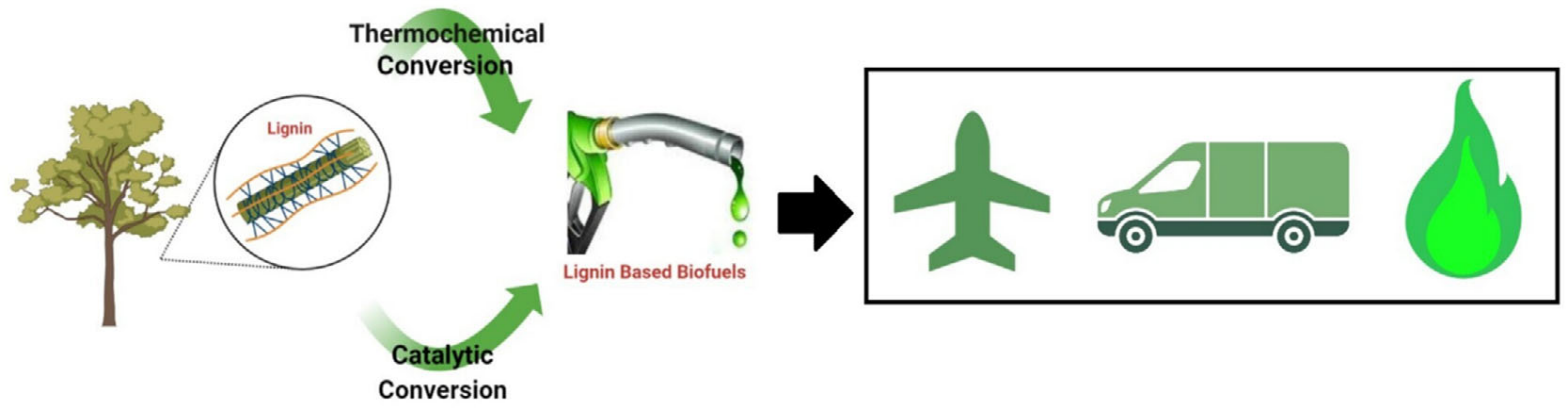
Lignin‐Based Biofuel Production. Reproduced with permission.^[^ ^114^ ^]^ Copyright 2023, licensed under CC BY 4.0.

Lignin, derived from LCB through various bioprocessing techniques, offers a versatile and sustainable resource with a wide range of applications. Some of these applications, previously discussed, include their use in sunscreen formulations,^[^ ^81^ ^]^ as a component in lignin‐phenol‐formaldehyde wood adhesive synthesis,^[^ ^77^ ^]^ as an antioxidant,^[^ ^67^, ^84^ ^]^ and for modifying LDPE polymeric membranes.^[^ ^60^ ^]^ More applications of lignin as a renewable feedstock for the production of high‐value products are considered in many of the recent studies and have been discussed in earlier reviews.^[^ ^108^, ^109^, ^110^, ^111^, ^112^ ^]^ The renewability and sustainability of abundant lignin made lignin a competitive alternative to fossil fuels in various fields such as polymer processing, fuels, adsorbents, synthesis of bio‐based products such as polyurethane foams, epoxy resins, carbon fibers, agriculture, and construction applications. **Figure** **11**. illustrates the potential applications of lignin.^[^ ^26^, ^113^ ^]^ Moreover, biolignin holds significant potential as a biofuel precursor due to its potential benefits, such as high carbon content, up to 60%, and energy density. Additionally, lignin is beneficial due to its renewability and carbon neutrality. It can be converted into biofuels through either thermochemical conversion or catalytic conversion, as shown in **Figure** **12**. These methods break down the complex lignin structure into simpler hydrocarbons, which can then be refined into bio‐oil, syngas, and other valuable fuels.^[^ ^111^ ^]^ Lignin can be converted into valuable biofuels using thermochemical methods, which are suitable for large‐scale production and offer economic benefits. Biomass, including lignin, can be gasified to produce a gas mixture of CO, H_2_, which is referred to as syngas. Pyrolysis converts lignin into bio‐oil, combustible gas, and biochar through heating at 400–750 °C. Moreover, lignin can be converted into valuable chemicals, such as aromatic compounds, which can be further processed into biofuels or used as additives to improve fuel properties.^[^ ^114^ ^]^

Biolignin has the potential to be used as an insulating material, such as polyurethane aerogels and other polymers. Petroleum has traditionally been the source of polyols used in polyurethane (PU) synthesis, which contributes to fossil fuel consumption and environmental pollution. However, lignin, which contains hydroxyl groups, presents a viable alternative as a substitute polyol for PU production. Consequently, several polyurethane (PU) polymers have been developed using lignin to either fully or partially replace petroleum‐based polyols.^[^ ^115^ ^]^ There are several methods for creating lignin‐based polyurethane, including either chemical modification of lignin or its direct integration.^[^ ^116^ ^]^ To enhance lignin's reactivity and suitability for polyurethane synthesis, various chemical modifications have been explored. These modifications include depolymerization, hydroxy‐alkylation, dealkylation, and esterification.^[^ ^117^ ^]^ Bio‐based rigid polyurethane foam (B‐RPF) was synthesized from extracted lignin from corn stover pretreated using an ethanol–water solution to extract lignin. The process selectively dissolves and precipitates lignin particles, producing a lower molar mass distribution and regulated structural composition. Oxypropylation with propylene oxide added aliphatic hydroxyl groups to fractionated lignin, to enhance the reactivity for polyurethane synthesis. The oxypropylated lignin fractions were used as biopolyols to replace commercial polyether polyol in B‐RPF synthesis. The modified lignin significantly improved foam performance and contained critical hydroxyl groups for polyurethane formation, indicating its suitability for B‐RPF production. B‐RPF meets insulation material demands (0.02 W m^−1^ K^−1^ to 0.05 W m^−1^ K^−1^), highlighting biolignin potential in bio‐based insulation applications.^[^ ^118^ ^]^

A novel approach to develop high‐performance bio‐based aerogels incorporating lignin, specifically Sodium lignosulfonate (LS) obtained from sulfite pulping liquors by Russia Paper Co., Ltd. Lignin incorporation in the aerogels caused significant improvements in mechanical strength as the incorporation of lignin enhances the aerogels’ compressive modulus by ≈ 30 times compared to lignin‐free aerogels. Lignin also served as a char‐forming agent due to its aromatic structure and high carbon content, thereby contributing to the flame retardancy of the aerogels. Additionally, the aerogels exhibit low thermal conductivity ranging from 0.046 W/(m k) to 0.037 W/(m k), highlighting that lignin incorporation enhanced the functional properties of aerogels, increasing its potential for various industrial applications, particularly in construction and transportation.^[^ ^119^ ^]^

Biolignin can also be utilized in construction and building materials, as it can be incorporated into concrete and other construction materials as a sustainable additive. It improves the strength, durability, and water resistance of the materials. The concrete water reducer, also referred to as a concrete plasticizer, holds significant importance in contemporary concrete compositions, as it plays a vital role in enhancing the workability of concrete. Its primary function involves reducing water requirements while simultaneously enhancing the strength and durability of the concrete.^[^ ^120^ ^]^ Pine wood lignin was employed to make a concrete water reducer. Lignin was extracted with formic acid and separated using organic solvents. Oxidation‐sulfomethylation (OS) modified fractionated lignin to study how fractionation affected the lignin‐based water reducer. After OS treatment, fractionated lignin using pure acetone had a higher sulfonation degree (SD) than unfractionated lignin. Cement pastes with fractionated lignin following OS modification had 21% more fluidity than sulfonated formic acid lignin without fractionation. Fractionated lignin has more reactive sites, such as methylene and phenolic hydroxyl groups, and fewer methoxyl groups. This increased reactivity improves lignin's water‐reducing properties.^[^ ^121^ ^]^

Biolignin can be used in carbon capture and storage (CCS) technologies as well. The successful utilization of black‐liquor lignin (BLL) to prepare porous carbon materials for CO_2_ capture was presented in research. Using pulping industry byproduct black liquor lignin, cost‐effective and stable adsorbents with abundant distribution have been developed. C‐BLL‐ZnCl_2_ and C‐BLL‐KOH target carbon materials were obtained by chemical activation with KOH or ZnCl_2_, followed by template method synthesis of porous carbon materials from lignin. The CO_2_ adsorption performance of lignin‐based porous carbon materials has been evaluated at various temperatures. The results showed that KOH activation can turn black liquor lignin into porous carbon compounds with strong CO_2_ adsorbent. The activation approach produced abundant disorder structures and greater BET surface area in carbon materials. Due to its micropore architectures and oxidized sulfur functional groups, C‐BLL‐KOH absorbed CO_2_ well, moreover, C‐BLL‐KOH also showed selectivity for CO_2_ adsorption in a CO_2_ and N_2_ combination, demonstrating the utility of using black liquor lignin as a feedstock for porous carbon CO_2_ adsorbents.^[^ ^122^ ^]^

Mulch films are commonly employed in agriculture to increase soil temperature, retain soil moisture, and enhance water and fertilizer efficiency. Biodegradable mulch films were synthesized utilizing lignin extracted from empty fruit bunches. The EFB supplied by the Suksomboon Group (Chonburi province, Thailand) underwent acid hydrolysis to eliminate hemicellulose and cellulose, facilitating the extraction of lignin, which was subsequently dissolved in Dimethyl sulfoxide (DMSO) and combined with PVA solutions at varying lignin concentrations of 0, 20, 40, 60, and 80% w/v. The mulch films were examined for opacity, biodegradability, water solubility, absorption, and mechanical characteristics. Additionally, a life cycle assessment (LCA) and cost estimation analysis were performed. The results indicated that an increase in lignin content enhanced the film's water solubility, moisture retention, and biodegradability. The LCA evaluations and cost analysis indicate that the lignin‐infused PVA film exhibited the least environmental impact and was more economical than the commercial mulching film. The findings indicated that blending polyvinyl alcohol polymer with lignin can enhance biodegradability by 25.47% through soil burial and 32% via water solubility.^[^ ^122^ ^]^

One of the many applications for lignin is its conversion into carbon fiber. Lignin‐based carbon fibers are extensively discussed in previous literature.^[^ ^124^, ^125^, ^126^ ^]^ A recent work demonstrated the effective synthesis of lignin‐based carbon nanofibers (LCNFs) as self‐supporting electrodes in supercapacitors and lithium‐ion batteries. Lignin was isolated from rice straw sourced from Hubei Province, China, utilizing ethanol. LCNFs were synthesized using an electrospinning technique followed by heat treatment. The effect of lignin addition on the morphology and structure of LCNFs was investigated, as well as their electrochemical performance as electrodes for supercapacitors (SCs) and lithium‐ion batteries (LIBs). Morphological and structural characterization demonstrated that LCNFs had a nanofiber crosslinked structure, a high specific surface area, a small average particle size, and a dense microporous network, all of which enhanced their performance in supercapacitor applications and as an anode material for lithium‐ion batteries (LIBs).^[^ ^127^ ^]^

Lignin's ability to participate in electrostatic interactions with bacterial cells and the presence of polyphenolic chemicals in lignin that undermine the integrity of bacterial cell walls encouraged the utilization of bio lignin as a beneficial material for demonstrating antibacterial effects.^[^ ^112^ ^]^ This study investigated rice straw valorization for lignin extraction as a precursor to biopolymeric films with improved antibacterial and biodegradability. Alkaline pretreatment isolated lignin, followed by acidification to precipitate it. Lignin with varying concentration was then added to solvent‐cast films by PVA and citric acid crosslinking. The antibacterial zone of inhibition against Pseudomonas sp. ranged from 0.23 to 4.1 cm and was directly related to lignin content. The films with the highest lignin content had the best bactericidal efficacy, while control films with no lignin did not have an inhibitory impact. This shows that these coatings can reduce bacterial contamination and preserve food taste. After being buried in soil, the films lost over 78.7% of their weight, outperforming nonbiodegradable synthetic polymers. These findings indicate a promising path for biopolymeric films as environmentally friendly food packaging alternatives to plastic wraps.^[^ ^128^ ^]^

### 4.2 Lignin Extraction and Utilization in Industry

The commercial advancement of lignin extraction started in Rothschild, Wisconsin, USA, in 1934 and saw notable expansion after 1971, resulting in the manufacture of new refined lignin grades. In the last ten years, the sector has advanced significantly in expanding commercially feasible lignin processes. Using the LignoBoost technique in a kraft pulp mill, the world's first large‐scale lignin production plant at New York, USA in 2013, marking a significant milestone. Under the BioChoice lignin name, 25,000 MT of lignin yearly is produced. A second LignoBoost plant was then started in 2015 at Stora Enso's Sunila mill (Finland) with a bigger capacity of 50,000 MT per year. Operating for more than 70 years, Borregaard's LignoTech in Norway tops sales of lignin‐based products with 366,000 MT reported in 2021, including its wood‐derived vanillin.^[^ ^129^ ^]^ Additionally, Pure Lignin Environmental Technology Ltd. (Canada), on the other hand, focuses on affordable lignin and cellulose extraction, providing a one‐of‐a‐kind water‐soluble lignin.^[^ ^130^ ^]^

Lignin's commercial uses are fast growing with ongoing developments, hence opening the path for a more sustainable future in various industries, including: 1) The world's first fully bio‐based furnishing board has been developed through the collaboration of Stora Enso and Koskisen. Koskisen, a plywood manufacturer, is the first company to utilize Stora Enso's bio‐based binder, NeoLigno, as a substitute for fossil‐based resins in furniture boards. The raw materials for the furniture board and the binder are obtained from the production processes of both companies. Consequently, all raw materials of the board are completely bio‐based.^[^ ^131^ ^]^ 2) A sustainable and environmentally friendly substitute for fossil‐based anodes in batteries. Lignode by Stora Enso is a bio‐based anode composed of hard carbon derived from lignin, a byproduct of the existing pulp industry. Lignode will transform the battery industry.^[^ ^132^ ^]^ 3) VITO, a sustainability research institute in Belgium, supported by the European Regional Development Fund (ERDF), collaborates with New Zealand's Scion to produce bioaromatics from lignin. VITO is currently engaged in numerous initiatives concerning the conversion of lignin and wood into bioaromatics: MAIA, ARBOREF, BIO‐HArT, SmartLi, and LigniOx. The pilot plant will have a production capacity of 200 kilograms per day.^[^ ^133^ ^]^ 4) Lignol represents a viable solution for achieving a fossil‐free transportation industry by 2030. RenFuel offers the technology for bio‐oil LIGNOL, which can be blended with any crude or vegetable oil and hydrotreated in a refinery to produce petrol, diesel, aviation, and marine fuels that are entirely compatible with conventional fuels. LIGNOL fuels provide a more economical price at the pump, greater feedstock availability, and significant greenhouse gas reduction.^[^ ^134^ ^]^ 5) Prisma has invented and patented technologies that generate many product categories, all centered on the conversion of modified and customized lignin to high‐value polymers and composite materials. Their first commercial product is a substitute material composed of ABS plastic, referred to as BioLAN. BioLAN is more cost‐effective, has superior tensile strength, enhanced UV resistance, and reduced VOC levels compared to conventional ABS resins.^[^ ^135^ ^]^

## 5 Economic Feasibility and Market Potential of Lignin Extraction

Evaluating the economic viability of lignin extraction techniques is crucial for establishing their industrial applicability and promoting further research and commercialization initiatives. Diverse extraction methodologies, encompassing traditional approaches such as kraft, soda, sulfite, and organosolv, alongside innovative green solvents including ILs and DESs, display unique cost frameworks and scaling disadvantages as mentioned in Table 1.

A comparative analysis of these approaches studied by Carvajal et. al indicated that soda and kraft extraction processes were the most economically viable due to their established industrial infrastructure and comparatively lower processing costs. Soda extraction was the most economical method, with a cost of ≈4.2 USD/kg of lignin when derived from rice husk, whereas the kraft process costs 4.8 USD/kg.^[^ ^136^ ^]^ Nonetheless, these methods frequently provide lignin of lower purity or undesired chemical modifications that restrict its utilization in high‐value industries. Organosolv extraction produces higher‐purity lignin, rendering it appropriate for advanced applications but with significantly higher costs, ranging from 7.2 to 10.7 USD/kg, depending on the biomass source. Expenses associated with solvent recovery pose economic challenges to widespread implementation.^[^ ^137^ ^]^

In advanced solvent‐based methodologies, particularly those employing ILs and DESs, economic viability is a significant issue. Although these solvents facilitate selective lignin extraction and enhance purity, their elevated operational costs and restricted recyclability constrain scaling.^[^ ^138^ ^]^ A techno‐economic assessment of industrial‐scale lignin fractionation plants estimates capital expenditures between 20 and 32 million USD, with operational costs varying from 390 to 460 USD per ton of lignin.^[^ ^139^ ^]^ Primary cost parameters encompass raw lignin acquisition, solvent utilization, energy expenditures, and maintenance. To improve the market potential of lignin‐derived products, additional research is required in the following critical domains: 1) Cost reduction through process optimization to reduce energy consumption and enhance solvent recyclability, 2) Partnering with industries in adhesives, construction materials, and specialty chemicals to create lignin‐based products that comply with regulatory and performance criteria. These collaborations may encourage widespread large‐scale adoption by assuring that lignin‐derived materials are competitive with petroleum‐based alternatives regarding cost, durability, and effectiveness; 3) Advancing economic modeling by evaluating the costs and benefits through a study of its economic and environmental effects. This involves evaluating production costs, potential profits associated with lignin‐based products production, and comparing their market value relative to existing materials. By solving these research gaps and enhancing interdisciplinary interactions among material scientists, chemical engineers, and industrial stakeholders, lignin can evolve from a byproduct to a commercially viable bio‐based resource.

## 6 Challenges and Roadmap for Future Research

### 6.1 Challenges

The major challenges and research gaps concluded from this review are elaborated below: 1) Enhancing extraction efficiency with combined approaches: The use of combination approaches, such as the combination of irradiation physical and chemical treatments, was found to be promising in addressing challenges by enhancing lignin extraction as lignin yield from bamboo biomass was 65.81 ± 2.40% using ultrasound‐assisted organosolv pretreatment showing an increase compared to the yield of 57.96 ± 3.76% without the ultrasound assistance.^[^ ^88^ ^]^ In addition, ultrasonic‐assisted alkaline treatment for rice straw reduced extraction time by 40% and increased yields from 72.8% to 84.7%, highlighting efficiency improvements.^[^ ^96^ ^]^ Further research is needed to optimize the irradiation parameters. 2) Green solvent recovery and economic feasibility: Some studies investigated the use of green solvents, such as DESs, with a noticeably high lignin yield of 82% using ChCl–urea for lignin extraction from SCB, as cited earlier.^[^ ^83^ ^]^ Also, organosolv pretreatment using ethanol for lignin extraction from bamboo yielded 65.81% lignin with 95.37 ± 1.17% purity.^[^ ^88^ ^]^ However, since solvent recovery is a critical factor for the economic feasibility of green solvent pretreatment, more research should focus on developing efficient recovery and recycling techniques. 3) Standardization of pretreatment and characterization methods: The lack of a standardized technique for lignin pretreatment, characterization, and yield calculations will be critical to expanding research and achieving the full potential of lignin extracted from LCB. By offering standardized methodologies and calculations, accurate comparison of results across research becomes possible, promoting innovation in lignin processing and assisting its widespread industrial use. Standardization will also help to develop lignin‐based materials in industries such as bio‐based materials, chemicals, and energy, driving the transition to a more sustainable, circular bioeconomy. 4) Scaling up extraction technologies for industrial applications: It is necessary to focus on scaling up these advanced extraction technologies for commercial viability. By addressing scalability challenges, including solvent recovery and process integration within existing biorefinery systems, the adoption of optimized methods and bio‐based solvents can lead to the widespread utilization of lignin across various industries.

### 6.2 Roadmap for Future Research

1) Investigation of optimal irradiation parameters, such as frequency, intensity, and duration, to study their effect on extraction efficiency. 2) Investigating solvent recovery using low‐energy separation methods (e.g., evaporation, membrane separation) to recover the solvents after pretreatment, which could significantly lower the operational costs of biomass processing. 3) Investigate new catalysts (e.g., metal salts, acids, or enzymes) for usage in green pretreatment methods, such as DESs, minimizing economic and environmental impact, while achieving higher yields, paving the way for more effective utilization of LCB in biorefinery applications. 4) Utilizing sophisticated computational tools, process to optimize lignin extraction processes through advanced software simulations and modeling holds to study the potential to enhance lignin's yield and quality. Researchers should fine‐tune critical parameters to ensure that extraction techniques are both efficient and effective. 5) Studying the impact of various catalysts on reducing temperature and pressure conditions during pretreatment. 6) Performing technoeconomic studies to investigate scaling up current lignin extraction methods to commercial levels while maintaining process efficiency.

Collectively, these efforts will contribute to the advancement of a circular bioeconomy, positioning lignin as a valuable resource for renewable materials and sustainable chemical production.

## 7 Conclusions

This article has provided a comprehensive review of the various methods for the extraction and utilization of lignin derived from diverse LCB. The review highlights that using a certain pretreatment method reveals significant variability in lignin yield and characteristics depending on the biomass type and pretreatment conditions; for instance, organosolv lignin extraction for oil palm EFB yielded high hydroxy phenolic content lignin, indicating its potential as an antioxidant for biodiesel applications.^[^ ^67^ ^]^ In comparison, SCB subjected to the same organosolv treatment yielded lignin with superior UV absorbance and strong antioxidant properties. These characteristics made it highly suitable for advanced applications, including UV‐protective sunscreens in cosmeceutical products.^[^ ^82^ ^]^

This emphasizes that the choice of extraction technique and biomass source significantly influences the yield and characteristics of lignin, which in turn affects its application potential. The review also emphasizes the challenges posed by lignin's distinct and recalcitrant structure, which necessitates the precise selection of pretreatment techniques tailored to specific biomass sources and intended applications.

## Conflict of Interest

The authors declare no conflict of interest.
